# The impact of the COVID-19 pandemic on frail older people ageing in place alone in two Italian cities: Functional limitations, care arrangements and available services

**DOI:** 10.1371/journal.pone.0298074

**Published:** 2024-03-15

**Authors:** Maria Gabriella Melchiorre, Stefania Cerea, Marco Socci, Giovanni Lamura

**Affiliations:** 1 Centre for Socio-Economic Research on Ageing, IRCCS INRCA—National Institute of Health and Science on Ageing, Ancona, Italy; 2 Social Policy Laboratory, Department of Architecture and Urban Studies, Polytechnic University of Milan, Milan, Italy; University of Naples - Parthenope: Universita degli Studi di Napoli Parthenope, ITALY

## Abstract

The study aimed to explore and compare effects of lockdown, due to the COVID-19 pandemic in 2020, on frail older people living alone at home in Brescia and Ancona, two urban cities located respectively in Northern and Central Italy. This country was the Western epicenter of the first wave of the pandemic (February-May 2020), which affected the two cities differently as for infections, with a more severe impact on the former. A follow-up study of the IN-AGE research project (2019) was carried out in July-September 2020, by means of telephone interviews, involving 41 respondents. Semi-structured questions focused on the effects of the first wave of the pandemic on their mobility and functional limitations, available care arrangements, and access to health services. The lockdown and social distancing measures overall negatively impacted on frail older people living alone, to a different extent in Ancona and Brescia, with a better resilience of home care services in Brescia, and a greater support from the family in Ancona, where however major problems in accessing health services also emerged. Even though the study was exploratory only, with a small sample that cannot be considered as representative of the population, and despite differences between the two cities, findings overall suggested that enhancing home care services, and supporting older people in accessing health services, could allow ageing in place, especially in emergency times.

## Introduction

Ageing in place has been defined as ‘one’s journey to maintain independence in one’s place of residence as well as to participate in one’s community’ [1]. It has also been highlighted [2] that ageing in place could allow maintaining autonomy and caring relationships with family and friends. However, older adults living alone at home, can be vulnerable to a different degree, depending on individual resources [3], in addition to how environment meets their needs [4]. The presence of a supportive environment [5] (e.g., availability of healthcare services, transport, social support, and opportunities of social participation) is crucial, particularly for frail older people with functional limitations [6], who are vulnerable to several illnesses and/or disabilities, and at higher risk of reporting dependency, hospitalizations and institutionalization [7]. Frailty is indeed a multidimensional syndrome affecting physical, psychological, and social domains of human functioning [8, 9], impacting in turn ageing in place. Previous authors have developed a conceptual model of ageing in place, by explaining why seniors prefer to live in own home in later life. In particular, Wiseman [10] put in evidence how ‘triggering push’ events, e.g., poor health/illness, need of help in the activities of daily living, income losses, and widowhood, make older persons less satisfied with their place of residence, thus motivating them to move elsewhere. Litwak and Longino [11] interpreted residential moving behaviours of older people more as socioemotional response to age-related life events, e.g., increased possibility to live closer to the family, following retirement.

In Italy the proportion of older people aged 65 years and over is about 24% of the total population as of 1^st^ January 2022 [12], about 50% of people living alone are over 65 [13], and 44% of those living alone have severe functional limitations [14]. In this context, ageing in place is mainly allowed by the family caregiving, especially wives and daughters (90%) [15], whereas the National Disability Attendance Allowance (*Indennità di accompagnamento*—IA) (see S1 Appendix for the full list of abbreviations) is provided to 12% of such population group, Home Care Service (social) (*Servizio di Assistenza Domiciliare*–SAD) reach only 1%, and the number of beds in residential care facilities is just 1.9 every 100 over 65s [16]. It is also to highlight that the cash-for-care scheme prevails in the Centre-South, e.g., Marche region, and the residential care in the North, e.g., Lombardy region [17]. Concerning private home care services, persons employed by families are mainly domestic home helpers (DHHs), and personal care assistants (PCAs), including migrant care workers (MCWs) when from abroad (e.g., Eastern Europe), often hired on an irregular basis, especially in the South [18, 19]. Further help (often free) comes from volunteering organisations [20]. Moreover, among older people, almost all contact the General Practitioner (GP), about 70% have visits from Medical Specialist (MS), and about 14% from rehabilitation professionals [14]. Overall, barriers in accessing these services derived mainly from long waiting lists in the public sector, and from the high cost in the private sector [21]. Such difficulties are more pronounced in the Centre than in the North of Italy [22].

A poor and/or inadequate care and cure network could make it difficult for seniors to remain at home, especially in a context of health emergency, as the one following the COVID-19 outbreak and the consequent lockdown. In this respect, since March 2020 stay-at-home and physical (or social) distancing measures, were adopted in almost all countries, often with an age-related approach [23], to reduce mobility and community transmission of the virus [24, 25]. These restrictions, i.e., non-pharmaceutical interventions (NPIs), were implemented by public authorities and imposed self-isolation, to avoid the spread of COVID-19 and protect the population, but were very difficult to enforce, and their effectiveness varied greatly among countries, also requiring voluntary compliance by citizens, and with characteristics of a country playing a key role in this respect [26]. In particular, social capital, defined as strong social interactions and family ties, can have a positive impact on the behaviour of a population during a pandemic, thus affecting its evolution and related effective respect of imposed government restrictions, with citizens complying more with NPIs in countries with higher social capital stocks, e.g., by maintaining social distancing and avoiding using of public transport [26]. More widely, both socioeconomic and psychological determinants impact the willingness and possibility to adopt protective behaviours and follow NPIs during a pandemic, including the context of social/family network and possible disrupted relations, socioeconomic status as available financial resources and health literacy, job insecurity and loss of income (especially when remote work/teleworking is not allowed), and overall fear for the pandemic [27]. Further authors indicate factors affecting compliance/appropriate behaviours with and effective implementation of NPIs, as follows: self-awareness of the citizens (in developed countries) with reduction in mobility mainly depending on their voluntariness and social responsibility [28]; positive attitudes and public beliefs regarding efficacy of various NPIs in reducing the risk of infection [29]; and high perceived level of risk associated with high level of NPIs adoption [30].

NPIs measures adopted by countries effectively reduced the transmission and slowed the increase of excess mortality [31], but also reduced social support and community services, increased economic difficulties, and delayed medical treatments, especially for older people [32, 33]. NPIs in Italy (and also in other countries, e.g., France and Germany) obliged persons to reduce the number of people allowed to visit relatives and friends in a private house, and this only once per day [26]. Moreover, the national lockdown imposed the closing down of businesses which were considered unnecessary [34], and allowed to go out of one’s house only for working, for reasons regarding health, and to purchase primary goods [35]. It is also worthy to highlight, that in Italy a strict lockdown was adopted in March 2020, then lifted during Summer 2020 following the reduction of new infection cases but also the pressure of some commercial activities loosing great earnings and protests of unemployed workers. However, in Autumn 2020 new infections were registered and further (even though lighter) NPIs were adopted, especially focusing school closure [34], Thus, in 2021, the movement “IoApro” (“I open” in English) initially asked the government to mitigate social distancing measures and re-open closed business activities, and then extended its anti-restriction approach to an anti-vaccine policy, whereas the Italian government imposed from April 2021 the so-called vaccine passport, in order to allow moving without restrictions only to vaccinated, or already infected and healed/immunized citizens, thus integrating pharmaceutical interventions (PIs) with NPIs [34].

Overall COVID-19 restrictions in Italy drastically reduced social interactions, especially with family members/relatives, friends, and neighbours, with hard consequences in particular on well-being of older people. This due to a forced isolation and ‘deprivation’ from possible support nets and caring supports [36], thus impacting the living situations of seniors, who were the individuals most vulnerable to the pandemic [37], and with the highest mortality risk due to COVID-19 [38]. Worldwide, more than 90% of recorded COVID‑19 related deaths in 2020 have indeed occurred among those aged 60 and over, and 58% among those aged 80 and over [39, 40], with a median age of 81 years [41]. Italy was the first European country affected by the COVID-19 pandemic, with one of the highest death rate from the virus, as recorded by the online scientific organization ‘Our World in Data’ (OWID) [42]. In spring–summer 2020, the cumulative COVID-19 confirmed cases per million people aged 50 years and over in the country, was 4,115.04, and the cumulative deaths per million people was 581.97 [43]. By the end of August 2021, the overall related mortality rate in Italy was about 35% higher than the European Union (EU) average [44]. In particular, the most relevant contribution to the excess mortality in 2021 in this country, respect to the average of the years 2015–2019, was due to the increase in deaths of the population aged 80 years and over, that explained 72% of the overall excess in mortality [44]. Overall, in the period 2020–2021, a total of 455,170 people aged 80 and over died in Italy (about 46,000 more than the average for the period 2015–2019) [45]. However, in Italy bonding social capital, as relationships and networks within own social group, had an important function in the greater capacity of some provinces in decreasing the pandemic escalation, suggesting the former as crucial for greater respect of the restrictions [26]. Moreover, socioeconomic determinants such as the structure of the local labour market (industrial/services vs agricultural workforce), high unemployment levels, higher education levels, population density (greater in urban areas vs rural), and demographic factors (e.g., old age), influenced the compliance with NPIs, leading to larger reduced mobility of citizens [46].

Following the above context, and defining frailty as age-related condition [8], in addition to living alone, with limited functional abilities in daily living activities [47], and needing for support [48], this study aimed to explore how frail older people ageing in place reacted to the first wave (February-May 2020) of the COVID-19 outbreak and the consequent lockdown, imposed by the national government for facing the spread of the epidemic. This in two Italian cities, Brescia (Lombardy region, Northern Italy) and Ancona (Marche region, Central Italy), in order to understand whether and how the pandemic impacted them to different degrees. The reason for choosing these two towns is double. On the one hand, the North of Italy can be considered as the Western epicenter of the first wave of the pandemic in February-May 2020 [49], with Lombardy region recording most of the cases in the first weeks of the health emergency, followed by Veneto, Piedmont, Emilia-Romagna, and Marche regions [50, 51]. In particular, in that period Brescia emerged as one of the Italian provinces most affected by the COVID-19 pandemic in terms of contagion and mortality rates, and the second at the regional level [52], whereas Ancona was less affected but however was the second province of Marche region recording the worst situation due to the pandemic [50]. On the other hand, this study is a 2020 follow-up of a previous main survey within the ‘Inclusive ageing in place’ (IN-AGE) research project [53], that was carried out in 2019 in some Italian urban/rural cities, including Brescia and Ancona, as explained better in the ‘Methods’ section.

In particular, the purpose of the paper is to answer to the following research questions: 1) Did the health emergency during the first wave of the COVID-19 pandemic impact on older people living alone, with regard to their functional limitations, e.g., mobility and other activities of daily living? 2) Did the pandemic impact on available care networks and services, e.g., family, friends and neighbours, municipal social services (e.g., SAD), DHH, PCA, and volunteering? 3) Did the health emergency due to the COVID-19 outbreak impact on access and use to/of health services? 4) Were there differences/similarities between the cities of Ancona and Brescia in this regard?

In this respect, we suppose that the pandemic and related lockdown could have caused hard consequences for seniors such as scarce mobility, thinning or loss of care networks, and problems for accessing health services. Moreover, due to the greater impact of the COVID-19 in Brescia province than Ancona, it could be expected also a worse impact of the social distancing on older people in Brescia city, as greater difficulty in meeting basic needs. The analysis of these aspects can be of help for understanding difficulties faced by older people living alone in case of health emergencies in two different areas of Italy, and which help was or was not specifically available during the pandemic, in addition to suggestions for possible interventions.

## Materials and methods

### Study design and data analysis of the main survey in 2019

The main survey, carried out in 2019, was part of a qualitative research project that involved 120 older people. In three Italian regions (Lombardy in the North, Marche in the Centre, and Calabria in the South), respectively three medium-sized urban cities [12] (with 100,000–200,000 inhabitants, i.e., Brescia, Ancona, and Reggio Calabria), and three inner/rural areas [54] were examined (respectively: Oltrepò Pavese, Appennino Basso Pesarese Anconetano, and Area Grecanica). Overall, the most fragile locations in such contexts were identified, e.g., with higher presence of older people living alone, share of households living in public housing (*Edilizia Residenziale Pubblica*—ERP), low level of education, low provision of public/essential services, socio-economic deprivation, high level of unemployment [55]. A purposive sampling was provided [56], with the aim of typological, rather than statistical representativeness, with characteristics of respondents allowing the analysis of the topics of the study, in addition to a theoretical generalization, as reflections for a discussion. Frail older persons were recruited through the local branches of a major volunteering organisation (i.e., AUSER—Voluntary association for active ageing), operators of municipal/public home care services (e.g., SAD), and other local/voluntary associations. The inclusion criteria were the following: men and women aged 65 years and over, living alone at home or with the help of a PCA, limited functionalities/mobility, absence of both cognitive impairment and of very close family members who give them support. Moreover, in order to reach a sampling saturation and develop preliminary theoretical categories of respondents, the following was decided: the same number of interviews in the different territorial contexts (40 per region, of which 24 in each of the three urban cities and 16 in each of the three inner areas); at least 20% of men, 20% with PCA, 30% with mobility only at home, and 25% with no help from the family.

Interviews were conducted face-to-face by using a semi-structured interview/topic guide, focused on socio-demographic aspects, family and housing, health status, daily living activities, use of services, care networks, social isolation and perceived loneliness. The topics were explored with ad hoc questions drawn and adapted from previous similar studies [57]. The limitations in performing the activities of daily life were detected by administering the Activities of Daily Living (ADLs) and Instrumental Activities of Daily Living (IADLs) scales [58], in addition to two sensory limitations (difficulties in seeing and hearing), and two mobility limitations (going up/down the stairs, and bending to pick up an object) [59, 60]. A written informed consent form was signed by each participant. The study was conducted in accordance with the Declaration of Helsinki, and the protocol was approved (for the whole Consortium) by the Ethics Committee of the Polytechnic of Milan (POLIMI), Support Services Area for Research and Didactic Innovation (Project identification code n. 5/2019, approved on 14 March, 2019). Participants were informed on privacy and anonymity of the information collected, according to ethical issues indicated by the EU General Data Protection Regulation (GDPR) n. 679, of 27 April 2016 [61].

Narratives were audio-recorded, transcribed in full/verbatim by interviewers and a qualitative analysis was provided following the Framework Analysis Technique [62], which includes reading the transcribed interviews, identification of categories/themes, indexing-labelling, construction of thematic chart, and interpretation of contents. Data saturation was achieved when, after reaching the total sample of 120 seniors, the progressive reading of the narratives indicated that further data were not necessary, since those already collected were consistent with the research questions and the overall typological representativeness mentioned above. In particular, a thematic content analysis was conducted [63], starting from the questions provided for the interview as preliminary conceptual framework [53, 64]. A manual qualitative analysis was performed, without the use of a dedicated software, as also supported by some literature [65, 66]. Qualitative dimensions were further quantified [67, 68], and more articulated quantitative classifications were provided, e.g., levels of physical limitations, and share of family help on total (as explained more in depth in the section below). Additional details on the methods of the main survey (Study Design, Data Collection, and Data Analysis), can be found in a previous publication [53], from which the section ‘Materials and methods’ has been partly adapted.

### The follow-up in 2020

#### The pandemic in the areas of investigation

In order to better contextualize the areas of investigation, some institutional data (mainly regional and provincial, since few local information were available) are reported. In Brescia province, in early June 2020, there were 1,200 total cases of contagion per 100,000 residents [69], with 28% of deaths on the total deaths, and a death rate of 200 per 100,000 inhabitants, attributable to COVID-19 [70]. The impact of the pandemic in Ancona province was quite different: the same values were much lower and respectively 400, 9% and 31. However, what united both provinces, and more generally what has characterized the pandemic everywhere, is the bad outcome mainly among frail older people. In Italy (February-November 2020), 60% of deaths due to COVID-19 involved the over 80 age group, with same percentages in Lombardy and Marche regions (62% in both) [49]. Moreover, about 84% of deaths from COVID-19 involved older people aged 70 and over in both regions, even though in Lombardy 47% of the total COVID-19 deaths were recorded, and only 3% in Marche region [49]. With reference to Brescia and Ancona cities, the information on the age and previous pathologies of the deceased from COVID-19 was fragmented, but the respective provincial data up to 1^st^ April 2020 already provided a rather clear picture. As for Brescia, the public data on 1,521 deaths in the period revealed that the average age of the deceased was 77.4 years, and that 87% had at least one previous disease [71]. As for Ancona, the data on 101 deaths revealed an average age of 79 years, and 95% of seniors with at least one previous pathology [72].

#### Sampling, data collection, and ethical issues

The follow-up study was carried out in July-September 2020, in relation to some dimensions investigated also in the main survey in 2019. This is a follow-up of a previous qualitative survey, and the sample size was determined by the same respondents available also in 2020, but it was however sufficient for the study’s objectives since only few seniors in 2020 were “lost”. Twenty and 21 older people were indeed interviewed respectively in Brescia and Ancona in 2020, compared to 24 in both cities during the previous survey in 2019. Respondents were re-contacted with the help of recruitment channels involved in the main research project (local branches of voluntary associations and operators of municipal social services, in particular home care, i.e., SAD). These channels activated the preliminary contacts to remind the interviewees of the IN-AGE survey in which they already participated, and to have a first potential adhesion to the second interview (then confirmed/communicated to the interviewers), related to the experience of pandemic and lockdown.

Interviews were realized by telephone because of social distancing measures imposed by the pandemic and implemented by the government. Thus, they were not audio-recorded, but answers were written on papers by interviewers, who were however the same who carried out the survey in 2019 in both cities. The questionnaire (S2 Appendix) was semi-structured with closed-ended questions, and the possibility however to specify/support the answers by means of free/open spaces in the questionnaire itself was also provided, as it was for the main survey. Questions addressed first of all the impact of the COVID-19 on health, e.g., having experienced or not a contagion. In addition, self-reported changes (compared to 2019) due to lockdown were explored with regard to the following aspects, which were already investigated in 2019, even though with less details (as explained better in the “Data analysis”) due to the limits of interviews by telephone: overall functional limitations, as mobility and ability to carry out the activities of daily living; available care networks/arrangements; and access/use to/of health services (GP, MS, other health services). It was decided to detect self-reported impact of the COVID-19 outbreak in order to catch the perceived changes on different domains of participants’ lives, by asking older people to report possible worsening/improving due to the pandemic [73].

Regarding ethical issues of the follow-up study, a query was sent to the POLIMI Ethical Committee that approved the main IN-AGE study (as described above), and a positive opinion was obtained (response 11 May 2020 to a query on the Authorization n. 5/2019), in order to carry out the interviews without a further new ethical approval, since the study framework was the same of 2019 (same subjects, contact and information procedure; questions regarding only changes with respect to answers given in 2019, apart from a general question on the potential contraction of the virus). Moreover, participants were not asked to sign an informed consent form, for obvious reasons due to the pandemic and consequent social distancing measures in place. However, the aim of the follow-up was fully explained and oral consent was obtained and documented by recruitment channels. In any case, reference was made to what was established by the EU Commission, which, in derogation from the GDPR n. 679/2016, did not require as mandatory the written informed consent from participants in surveys conducted during the first phase of the COVID-19 emergency, and regarding the impact of pandemic on the population. This because such studies were considered of significant public health interest, and thus also conducive with simplified/emergency procedures [74].

### Data analysis

A simple quantitative analysis (frequency distribution/bivariate analysis) of closed responses was carried out by using Microsoft Excel software 2019 (Microsoft Corporation, Washington, USA).

Physical/functional limitations have been classified for T1 as follows [75]: mild: no activity ‘not able’; moderate: one-two; high: three-four; very high: five and more. Also, with regard to care arrangements, at T1 three shares of family support on total available help, including friends, neighbours and services, were calculated: no family help; moderate family help up to 50%; strong family help over 50%. These classifications were not provided for T2, because the COVID-19 emergency allowed only short telephone interviews, without the possibility to collect more detailed information as for the 2019 survey, through in-depth face to face interviews (for instance, by administering ADLs and IADLs scales, and through in depth questions on who/when/how much/for what was providing support to the respondents). Thus, functional limitations and care arrangements for T2 have been explored only as changes referred directly by respondents compared to T1, and regarding: general mobility, ability to carry out as a whole the activities of daily living (washing, preparing food, cleaning the house, shopping), and source of support (from family, friends, neighbours, and services). Responses for T2 (changes) have however been linked to respective classifications elaborated at T1.

The access to health services has been analyzed comparing the use referred at T1 with regard to GP, MS, and other health services (e.g., rehabilitation/physiotherapy, nursing services, diagnostic tests mainly in hospital), both from public and private sectors, with possible different/worsened access to these three services at T2, as referred by respondents (for prescription of drugs, therapies, clinical examinations, physiotherapy, and surgical interventions).

In all tables presented below, at T1 only respondents who participated also in the follow-up study at T2 are included, apart from Table 1, whose aim is to present the full sample at T1 and the (slightly) smaller sample at T2. Tables also show only absolute values (and not percentages), due to the small sample size (n = 41). Moreover, several empty spaces in the tables reflect the choose to show only the answers of participants referring changes.

**Table 1:** Sample characteristics: T1 survey (2019) and T2 follow-up (2020) (absolute values/n).

Characteristics	Brescia		Ancona	
	(Lombardy)		(Marche)	
	T1	T2	T1	T2
**Age groups (years)**				
65–74	5	5	3	3
75–79	4	4	4	3
80–84	6	5	5	5
85 and over	9	6	12	10
**Gender**				
Male	5	4	5	4
Female	19	16	19	17
**Education**				
No title	1	1	2	2
Primary school (5 years)	10	10	10	9
Middle school (3 years)	5	5	7	5
High school (3–5 years)	8	4	5	5
University/similar (3–5 years)	-	-	-	-
**Marital status**				
Single	5	4	2	2
Married but not cohabiting	-	-	1	1
Divorced/separated	7	7	2	2
Widowed	12	9	19	16
**Living situation**				
Alone	24	20	21	17
With cohabitant PCA ^a^	-	-	1	3
With hourly PCA ^b^	-	-	2	1
**Total respondents**	24	20	24	21

^a^ Cohabitant Personal Care Assistant (PCA)

^b^ Daily/nightly regular attendance for at least 28–30 hours a week.

Open responses were not categorized, unlike the main survey in 2019, when conversely a thematic content analysis was carried out in this regard, since free narratives were really few, fragmented, and overall supporting the closed answers. However, along the results section, some short quotations have been included when relevant (where BS stands for Brescia, and AN for Ancona). Further details have been added within the text, by means of simple quantifications/count of things, when reported as spontaneous/not asked narratives [76], even though by few interviewees, with the aim to include other information integrating the overall findings, e.g., worsening due to reduced/suspended SAD/MS visits; specific family members (e.g., daughter) providing particular help during the lockdown.

Overall, the research team has a deep experience in qualitative research and data collection/analysis, with a background as psychologist, sociologist, and gerontologist. Also, all have extended involvement in studies regarding the issues of ageing in place and caregiving of older people. Moreover, some team members are health services workers, with expertise in overall health-related aspects of seniors.

## Results

### Socio-demographic characteristics of the sample

The follow-up could not include only four subjects in Brescia (three deceased before the start of the follow-up in July 2020, and one refusal to be interviewed) and three in Ancona (one deceased and two refusals), thus the main socio-demographic characteristics of the samples at T1 [42] and T2 were essentially the same. Overall, the following prevail: older people aged 85 and over (especially in Ancona), women, with a low level of education, widowers (particularly in Ancona), and living alone without PCA (only in Brescia) (Table 1).

It is worth to highlight that recruitment channels/families reported how four seniors who were interviewed in the survey carried out in 2019, deceased before the follow-up (as anticipated above), for reasons different from COVID-19 pandemic. Also, during the period July-September 2020, no participant died due to virus contagion.

### Functional limitations: Mobility and other activities of daily living

At T2 only a woman in Brescia referred to have contracted the COVID-19 virus. Apart from this case, with physical/functional capacities compromised by the infection, as it was supposed nobody reported an improvement, whereas mainly poor mobility and other physical consequences following the lockdown and stay-at-home measures emerged, with considerable differences observed between the two cities (Table 2).

**Table 2:** Functional limitations at T1 and self-reported worsening at T2 (absolute values/n).

Level of limitations ^a^	Brescia			Ancona		
	(Lombardy)			(Marche)		
	Limitations	Mobility ^b^	Other ^c^	Limitations	Mobility ^b^	Other ^c^
	T1	T2	T2	T1	T2	T2
Mild	9	1	2	4	1	2
Moderate	4	1	-	6	3	2
High	5	-	-	6	5	5
Very high	2	-	-	5	5	4
Total respondents	20	2	2	21	14	13

^a^ Mild = no activities ‘not able’, Moderate = one-two, High = three-four, Very High = five or more

^b^ Mobility in general at T2

^c^ Other functions on the whole at T2: dressing/undressing, washing hands/face, bathing/showering, preparing/eating/cutting food, cleaning the house, washing the laundry, shopping, taking medication, managing finances. In some case worsening both in mobility and other functions were reported.

On the whole, in Brescia a worsening of physical limitations in four out of 20 cases were reported at T2 (respondents with mild/moderate limitations at T1). Such worsening regarded the general mobility and other abilities requiring walking outside the home (e.g., go shopping for procuring food and drugs and/or go to the pharmacy for getting medicines), since stay-at-home constraints and low physical movement due to lockdown negatively affected the overall capacity of respondents in this respect:

*When I left the house to go to the pharmacy, I had a hard time standing and waiting in line. I got tired right away. (BS-16)*

In Ancona, in over half of the cases a worsening both in the ability to perform alone the various activities of daily living, and concerning the overall mobility, was referred (mainly by those with high/very high functional limitations at T1):

*I already had problems walking before the pandemic, with the lockdown I got worse, I was blocked. Before I used to go for a walk every day! (AN-16).*

### Care arrangements

Results regarding different care arrangements (as diverse sources of support/help provided to respondents) are reported in Table 3.

**Table 3:** Help provided from family, friends/neighbours, municipal social service, DHH, PCA, and volunteering at T1, and self-reported changes in help at T2 (absolute values/n).

Help provided	Brescia			Ancona		
	(Lombardy)			(Marche)		
	Help	Worsened	Improved	Help	Worsened	Improved
	T1	T2	T2	T1	T2	T2
**Share of family help** ^**a**^						
No help	8	-	1	5	-	-
Moderate	9	3	1	12	1	7
Strong	3	-	1	4	2	1
Total respondents	20	3	3	21	3	8
**Friends/Neighbours**						
No help	16	-	1	10	1^b^	-
Help	4	-	2	11	2	4
Total respondents	20	-	3	21	3	4
**Municipal social services**						
No help	8	-	-	10	-	1^c^
Home care (SAD)	12	2	2	9	8	-
Day centre	-	-	-	2	2	-
Total respondents	20	2	2	21	10	1
**DHH**						
No help	10	-	-	9	-	1^d^
Help	10	4	1	12	7	2
Total respondents	20	4	1	21	7	3
**PCA**						
No help	20	-	-	19	-	1
Help	-	-	-	3	-	1
Total respondents	20	-	-	21	-	2
**Volunteering**						
No help	20	-	-	17	-	-
Help	-	-	-	4	2	-
Total respondents	20	-	-	21	2	-

^a^ Share of family help = number of family members who help on the total help (from family, friends/neighbours, public services, private services, DHHs, PCAs, and volunteering). Moderate family help = up to 50% of the total; Strong family help = over 50% of the total

^b^ In 2019 the interviewee had no help from friends/neighbours, but the presence of new neighbours, with whom she had no confidence, was reported as a worsening

^c^ An interviewee, who in 2019 had not reported the support from SAD, refers that during the lockdown the municipal social service operators contacted her to verify possible needs

^d^ An interviewee, who in 2019 had not reported the support from DHH, refers it during the follow-up.

With regard to family support during the lockdown (Table 3), in Brescia three respondents (with a moderate family help at T1) reported a worsening. Three seniors referred an improvement about help provided from the children:

*My daughter has come to do the heavy housework for me. (BS-12)*

In Ancona, the support from the family (greater than Brescia since T1, with 16 cases vs 12) (Table 3) changed in more than half of the cases during the follow-up, and situations of improvement prevailed, regarding mainly people who referred a moderate family support at T1. Three cases of worsening in Ancona regarded children living abroad. As of the improved situations, these involved children, brothers/sisters or grandchildren, who supported their relatives during the first wave of the pandemic with greater assiduity than T1, by delivering food and medicines at home:

*My family members have always been very present, and have supported me in all my needs in this period, also more than before the pandemic. (AN-15)*

Support provided by friends and neighbours (Table 3) is presented together since in later life these sources of supports often coincide, as also confirmed by respondents. Such support at T1 was more common in Ancona than Brescia (11 cases vs four). At T2 this source of help in Ancona worsened and improved almost in equal measure. The worsening regarded the reduction in contacts with, and related supports from, friends/neighbours, for fear of contracting the virus. When this support improved, more present/available friends, and little courtesies provided between neighbours, were generically reported. In Brescia, the help of friends and neighbours only improved (three cases):

*A neighbour of mine did some shopping for me, and I did the same for her. We helped each other, in short. (BS-20)*

*When I needed anything, I could call my neighbours. (AN-18)*

The support from municipal social services (home-based and semi-residential) (Table 3), to a substantially similar extent in the two cities was used at T1 (12 cases in Brescia, and 11 in Ancona). During the first wave of the pandemic, these services do not seem to have undergone particular changes in Brescia, where SAD was never reported as interrupted. In fact, only in two cases (out of 12 who used it at T1) these services were defined as worsened due to a reduced provision frequency, and not with regard to the quality:

*SAD operators did not come like they used to, so once I took a bath by myself. (BS-17)*

Conversely, a worsening of the municipal social services (SAD and day centre) for 10 seniors at T2 in Ancona was reported, following the suspension (reduction only in one case) during the lockdown:

*I used to have SAD three to four times a week, now sadly it has been suspended! (AN-20)*

It should however be emphasized that, in three cases in which SAD was suspended in Ancona, the social workers and the service operators were still present, as much as possible, for the essential needs, with home delivery of food and drugs at least.

Even the support from DHHs was provided to a similar extent in both cities at T1 (Table 3). At T2, this support changed in Brescia in half of cases among those who were users at T1. In four cases it was reported a worsening because of the suspension or reduced frequency of the service provided, due to the will of older people/their relatives, or of the domestic helper, to reduce the risk of infection:

*Before the emergency, the DHH provided heaviest housework for me once a week. Now she comes when she can. (BS-15)*

However, in Ancona a worsening in this kind of support has been most frequently observed. In seven out of 12 cases the service indeed failed (two hourly reductions and five suspensions) for precautionary reasons, but on the other hand, there was an improvement for three older people, with a greater hourly commitment of the DHH. When this source of help worsened, seniors tried to do by themselves:

*The fear of contagion prompted me to get by, to do certain household tasks by myself. (AN-3)*

Only older people interviewed in Ancona reported to have received help from PCAs and volunteers at T2, and to a marginal extent (Table 3). In Brescia, such aids were not present at T1, and were not activated even during the first wave of the pandemic. Regarding Ancona, three older people with PCA were detected at T1 (one cohabitant and two on an hourly basis), four at T2 (three cohabitants and one hourly) (Table 1), and two improvements at T2 were reported (Table 3), following a PCA who moved from hourly to in-house asset, and a new hired cohabitant PCA:

*I hired a Romanian cohabitant PCA, I needed more help in this hard period! (AN-10)*

Moreover, in Ancona four older people took advantage of volunteering at T1, and of these two reported a worsening at T2, due to the interruption of these services during the lockdown, in particular for accompaniment and transport:

*Volunteers suspended the transport service for medical visits, this was a great problem! (AN-5)*

### Access to health services

Concerning health services, i.e., their use and access, it was observed a further impact of the pandemic on older people (Table 4).

**Table 4:** Use of health services at T1 and self-reported changes in access at T2 (absolute values/n).

Health Services	Brescia			Ancona		
	(Lombardy)			(Marche)		
	Use	Different	Worsened	Use	Different	Worsened
	T1	T2	T2	T1	T2	T2
GP ^a^	20	3	4	21	12	4
MS ^b^	15	1	6	19	-	9
Other ^c^	6	-	1	11	1	7
Total respondents	41^d^	4	11	51^d^	13	20

^a^ General Practitioner (GP)

^b^ Medical Specialist (MS), both private and public

^c^ Other health services, both private and public: nurse, rehabilitation/physiotherapy, diagnostic tests

^d^ More health services were used in several cases.

With regard to T1, all the interviewees used the GP, but also the MS and other health services (e.g., rehabilitation/physiotherapy, nursing services, diagnostic tests mainly in hospital) were fairly frequently requested, especially in Ancona. At T2 access to both GP and MS (for prescription of drugs and therapies, clinical examinations, check-up regarding pathologies, etc.), in addition to other health services, revealed changes and adaptations faced by older people in both cities, most of which referred as worsening.

In Brescia a total of 11 worsening cases were reported, and a ‘different modality’ in access only in four cases. As for the GP, the suspension of outpatient visits, and the main practice of telephone contacts, were experienced as a simple change in accessing the service. Relations with the GP were conversely reported as worsened in four cases, following the unavailability of home visits and the long waiting time for collecting prescriptions and medical referrals. Six older people reported a worsening because several MSs suspended their visits:

*I had to wait a lot of time for receiving prescriptions from my GP! (BS-14)*

*I was unable to make the annual visits to the cardiologist and orthopaedist because they were suspended. (BS-8)*

However, the hardest context was found in Ancona, where a total of 20 worsening cases were referred. Four (as in Brescia) regarded the GP, who was very difficult to contact and to receive at home. Moreover, nine cases of worsening regarded MSs, and seven (vs just one in Brescia) other health services, due to the suspension by the provider, or the renunciation of older people for fear of contracting the virus:

*The GP never came home during the lockdown. It is evident that he was more afraid than me! (AN-1)*

*I gave up cardiologist check-ups for fear of COVID-19, to avoid dangerous situations, for fear of getting sick. (AN-4)*

*I had to give up physiotherapy in the hospital since this service was suspended. (AN-21)*

Twelve respondents in Ancona (vs three in Brescia) also reported a different way of accessing GP services, not considered as worsening (e.g., contacts through family members and/or using e-mail):

*To contact my GP, I had to ask my daughter for help, but this was not a great problem. (AN-8)*

## Discussion

The aim of this study was to explore effects of lockdown, due to the first wave of the COVID-19 pandemic in 2020, on frail older people living alone at home in the cities of Brescia (Northern Italy) and Ancona (Central Italy). Even though the sample is very small, the study offers some inputs to be considered and discussed, since the survey was carried out during the COVID-19 pandemic, that is a crucial period of health emergency hardly impacting the seniors, and in which to carry out surveys was quite challenging, so that evidence collected can contribute to integrate knowledge on the issues investigated. Moreover, despite boundaries hampering a precise scientific evaluation of results, the occasion of the follow-up permitted to re-contact older people already interviewed in 2019, to make them feel our closeness, and to collect their experiences and needs. Also, the interviewees themselves appreciated being contacted again, thus feeling less alone. Findings showed overall that seniors had a stronger support from home care services in Brescia, and a greater support from the family members in Ancona, where access to health services emerged as more problematic. The comparison between the two urban contexts also partly highlighted regional welfare inequalities in Italy which impact on ageing in place, as discussed below. It is to premise that general data (international/national, and regional) to discuss research findings are also reported, since little local information on Brescia and Ancona were available. It is also worth to highlight that in Italy the socio-health response to COVID-19 pandemic was implemented mainly at the regional level.

### Functional limitations

The follow-up interviews revealed an aggravation of physical/functional limitations, which mainly impacted respondents in Ancona than Brescia. On the whole, older people who reported a worsening of mobility, attributed it to the forced and prolonged stay-at-home during the lockdown, a circumstance that negatively affected walking and postural balance (as well as mental health and well-being). According to ISTAT [77], in 2020 the use of protective face masks was reported by 90% of the Italian population aged 18 and over, but only by 73% of the over 75, probably indicating that older people went out more sporadically during the lockdown. The worsening in the functionalities of daily living (e.g., shopping), which mainly affected respondent living in Ancona, was in turn influenced both by the reduced mobility and the great difficulty in performing them without help, especially if a support in this respect was available before the pandemic. As also pointed out by Age Platform Europe [78], especially older people living alone had major difficulties in accessing essential services during the pandemic, such as purchasing food and to get necessary drugs. Other authors [79] highlighted how the forced physical/social distancing imposed by the health emergency, often represented an additional fatigue for older people, especially if some relatives or friends could no longer meet their needs, at least partly. Moreover, following the restrictions imposed during the lockdown, older people have sometimes avoided going out, due to fear of contracting the virus [80], thus indicating also a worsening of the ‘psychological’ ability to move, besides the physical one. This happened also in Brescia and Ancona. However, a rather different starting context at T1 for the two cities should also be considered, with a greater presence of serious limitations in Ancona compared to Brescia. Thus, in Ancona, the worsening in physical functionalities mainly affected those with high/very high limitations at T1, almost to indicate that the forced inactivity worsened functional/clinical situations already precarious. Besides COVID-19, it is anyway to highlight that in later life, multimorbidity itself is often associated with a decline in functional capacity and mobility [81]. Conversely in Brescia, older respondents who reported a decline in physical limitations at T2, referred mild/moderate frailties at T1. Probably, and with particular regard to mobility (transversal aspect of all activities of daily living in some way), these subjects went out home more often than others at T1, even if supported by an aid in some case. The restrictions imposed by the lockdown, and also the fear of getting sick, worsened their conditions, because probably deprived them of a minimum physical exercise allowing to maintain some residual movement capacities.

### Care arrangements

In Italy, the care of older people ageing in place is mainly managed by family members, who represent the pillar of the Italian welfare system, for traditional cultural reasons and also because of the scarce diffusion of home care services, especially public ones [16, 82]. The pandemic hardly impacted this asset, with consequences in terms of further/exacerbating difficulties in delivering care services, especially during the lockdown, and greater criticality of some existing gaps in this respect, such as the fragmentation and regionalization of the whole socio-health care system [83], with differentiated regulations in identical services within the country, and better/more provision in the North [84].

The IN-AGE study in 2019 highlighted a greater family support in Marche region than in Lombardy at T1 (83% vs 68% of cases), whereas public services (e.g., SAD) sustained older people to a similar low extent in both regions (28% vs 30%) [53]. With specific regard to Brescia and Ancona, the support of municipal social services emerged as enjoyed by a substantially equal number of older people in both cities, while the presence of family members in the care networks was higher in Ancona. In view of this, the follow-up study in 2020 revealed a greater overall stability of care networks in Brescia, especially of municipal social services, and a greater involvement of families in Ancona. In Brescia, municipal social services (e.g., SAD) responded much better to the emergency, with some sporadic reduction in the provision frequency, whereas in Ancona there was the temporary suspension (reduction in only one case) of this service and closure of the municipal day centre. It should be noted that Marche region [85, 86] did not officially suspend the SAD with regulatory provisions, but the need to support *in primis* the most severe cases, and the scarcity of protective devices for home care workers, negatively impacted de facto the delivery of this service, especially in the initial phase of the first pandemic wave (February-May 2020) [84, 87]. Despite this, as reported by the interviewees, social workers on their own initiative kept in contact with users and tried to overcome the lack of services, to meet urgent needs as much as possible. A better context in Lombardy emerged, where in March 2020 various measures were adopted to deal with the health-social emergency. Additional resources were indeed used for expanding the delivery of SAD, especially in situations of extreme frailty, by means of additional/emergency home delivery of food and drugs [88]. Furthermore, for older people without support networks, the meals on wheels were enhanced, and shopping vouchers for basic needs (food, personal and home hygiene) were provided [89]. However, in Ancona more than Brescia, the follow-up study recorded cases of improvement as increased family support (respectively, eight vs three). The role of family members was thus accentuated in the former city, probably to replace other decreased supports, especially public home care services.

With regard to family support, data from the *Survey of Health*, *Ageing and Retirement in Europe COVID-19 period* (SHARE-COVID-19, across 23 EU countries and regarding population aged over 50) [43] showed overall that adult children were the effective pillars in supporting older parents in almost all countries, in addition to neighbours and friends. Moreover, the same data highlighted overall how informal care was in some case more resilient than formal/public home care services, with seniors reporting several difficulties in receiving the latter during the pandemic. In Italy, after the COVID-19 outbreak, the share of population reporting such difficulties was 30% (EU average 19%). Such a general context is underlined also by Age Platform Europe [78], that revealed an additional burden for informal caregivers/family members who provide assistance to their older relatives, due to the temporary closure of day care centres, the suspended/reduced/postponed availability of home public services, and the lack of DHHs. Therefore, even though on the one side a greater support from family was an improvement for the seniors, on the other side family could have managed a considerable, and in any case unexpected, increase in caregiving. In relation to this context, some authors [33, 90] put in evidence that the lockdown increased family carers’ burden, and caregivers felt greatly the hard impact of the pandemic on their care responsibilities, especially when the cared for suffered from dementia. According to ISTAT [77], during the lockdown, about 24% of the Italian population aged 18 and over received visits mainly from relatives, who brought them food and drugs. However, these percentages rise to 32% in the 65–74 age group, and to 60% among the over 75. It is furthermore to highlight that some literature indicates how relatives, when present, provide more support when negative/tragic events (e.g., the loss of a relative, a fall) occur in the life of older persons [91], thus impacting positively on their resilience, especially when they are frail and disabled [92].

### Access to health services

The COVID-19 pandemic significantly disrupted access to non-COVID-19 care services for many patients in all EU countries, especially seniors who avoided using health services, for fear of contracting the virus, but also due to a temporary decline or suspension of non-urgent/non-COVID-19 care services, with a consequent reduction of visits/treatments for non-urgent complaints different from the pandemic infection [44, 93]. Also, mortality for non-COVID illnesses increased, and hospitalizations decreased [94]. During the first 12 months of the pandemic, in Italy overall 23% of population reported having given up on cure (EU average 21%) [41], with psychological fear of contagion playing a key role [93].

This general context is reflected also in our findings regarding two Italian cities, where the pandemic strongly reduced the possibility of accessing health services (GPs, MSs, and other services). In this regard, the follow-up study revealed a more problematic context in Ancona, especially regarding MSs and other health services, whereas a similar worsened access to GP in both cities was mainly attributed to the suspension of home visits, following the precautions adopted during the first phase of the infection, when in any case, protective devices were lacking also for health professionals [95]. *Cittadinanzattiva* [96] underlined that this suspension impacted more on older people and patients with chronic conditions, with an increase feeling of being neglected and abandoned by GPs. Kuper and Shakespeare [97] highlighted that in general people with disabilities seem excluded from an appropriate pandemic response, thus amplifying a neglect that was already existing. It is anyway to put in evidence that, in both cities, the pandemic emergency heavily involved GPs and MSs in the management of the infection [98], and this generated an overload of COVID-19 and non-COVID-19 patients to be cured, with reduced possibility of assisting the latter adequately, with regard to both health and social needs [88]. Also, workload and distress of healthcare professionals in the period affected the relationship with patients [99]. In some cases, the use of consultations by phone or e-mail was adopted and accepted. In this respect, such different channels of accessing the GP’s services was reported mainly in Ancona than Brescia, probably following a custom already existing in the latter. Overall, in most EU health systems, primary care services replaced face-to-face consultations, in some cases also with the use of telehealth services, during the pandemic, in order to protect both patients and health care workers from the infection [44]. When no changes in accessing health services emerged in both cities, probably there was no need for them during the lockdown, whereas it seems less probable that such services were used as before the pandemic without particular problems.

The overall better situation in Brescia than Ancona, also reflects the pre-pandemic difference between the respective Regional Health Systems (RHSs). The Italian National Health Service (NHS) presents indeed historical ‘distortions’, which have compromised its effectiveness in emergency management, mainly due to a ‘differentiated regionalism’ generating welfare territorial inequalities, especially regarding access to health services [100]. As Spandonaro and D’Angela [101] underlined, in Italy three clusters of RHSs can be identified, based on levels of performance: excellent for five regions, including Lombardy; intermediate for 11 regions, including Marche; and critical for other regions. Furthermore, according to data from the 32^nd^ EURISPES report [102], public health expenditure in Italy is higher in the North, with greater values e.g., in Lombardy, where health system is described as one of the best in the country, thus allowing to offer high-level health services. However, the health system in Lombardy faced a stress test during the lockdown. Over the last years, this region has indeed implemented a health policy increasingly centred on both large hospitals and the private market [103]. The excellence of the hospital system and specialist medicine have in turn weakened the asset of territorial assistance, in reason of strong long-lasting disinvestments in this respect, with difficulties in dealing with the pandemic health emergency, and heavy consequences on the support to be provided to COVID-19 patients, especially older people [104]. Thus, in Italy the pandemic led overall to saturation of hospitals and acceleration in deaths, mainly of older people, despite a well-developed healthcare system in several affected regions, especially in the North of the country [44].

### Limitations

The follow-up study has some limitations to be considered. First of all, the paper is based on a small sample size (total N = 41) that cannot be considered representative of the target population, this limiting the generalizability of the findings. Further aspects impacting the degree to which the findings can be generalized regard the limited areas of investigations (only two cities), the answers based on self-reports by older participants, and the exclusion of seniors with cognitive impairment (not able to appropriately complete the survey). All this thus allowing only simple/general conclusions and purposes from the study, rather than politically significant insights. Also, absolute values in tables should be interpreted with carefulness, since they are very low. The interviews were short and carried out by telephone, since face-to-face administration was prevented during the lockdown due to the social distancing imposed by the pandemic and related government restrictions, without the possibility to collect detailed audio-recorded narratives as for T1 (replaced at T2 by written responses/notes that interviewers filled-in during the short call phone). As a consequence of this, only short responses and few verbatim quotations were available/relevant and reported in the text to support the findings. Also, self-reported changes at T2 due to the pandemic (worsening/improving) with regard to T1, rather than direct measurement as it was at T1, represents a limitation that in turn did not allow specific statistical analysis, e.g., regarding very small samples of repeated measurements or longitudinal data [105]. Moreover, when interpreting the follow-up results, it should be considered that for some dimensions (e.g., ability to carry out the activities of daily living), functional declines might be a consequence of ageing or previous precarious health conditions, and thus not clearly linked to the COVID-19 pandemic and related lockdown, due also to the difficulty, for older respondents, in differentiating such circumstances. Furthermore, the overall inclusion/comparison of data at different territorial levels (e.g., municipal with regard to results of follow-up, provincial with regard to infections and deaths for COVID-19, and also regional/national/international for their discussion), is sometimes depending on the paucity of information provided by available sources, which were however mainly in Italian, especially when regarding local data. For this reason, the manuscript contains several references in this language. A further limitation is linked to the sample composition in 2019/T1, that is mainly composed by women than men (90 vs 30), and that is in turn reflected in the sample for Ancona and Brescia in the same year (19 vs 15 in both cities). This did not allow the exploration of the gender dimension, even though this could overall provide further inputs. Additional limitations concern the survey carried out in 2019/T1, as follows: subjects were selected for their typological and not statistical representativeness, since the study was more qualitative [106]; it involved only three Italian regions, which cannot represent the whole Italian context, even though they may be considered representative of different degree of socio-economic development in Italy; the definition of frailty is limited to old age (65 years and over), ageing alone in place, and presence of functional limitations needing support in the activities of daily living; the cognitive assessment of interviewees was based on the information from the recruitment channels, then confirmed by the respective families. It is finally to highlight the necessity to repeat such follow-up studies annually, to truly be useful to the Italian community, in order to know how the situation of participants changed or not after the first follow-up (July-September 2020), e.g., seniors who died or faced several increasing difficulties in meeting their needs. Unfortunately, no further follow-up was carried out, and thus the resilience or ability of these seniors to survive in such situations, especially those living alone without support, was not further explored. This should be considered an additional limitation of our study.

### Suggestions for possible interventions

Despite the limitations mentioned above, our findings overall might suggest some possible interventions for frail older people. Our results have in particular provided a picture of risks and needs in supporting an ageing population during a pandemic, and might offer insights also for preventing and managing emergencies, even in the future. To this end, a preliminary and necessary step is the development of system actions, i.e. regulatory, legislative and financing interventions, for the redefinition of the governance of the complex of services involved in taking care of frail older people. In this respect, it is important to overcome the lacking integration between health and social service systems in Italy [98], with healthcare currently centred on the provision of health services, without including and integrating social ones, especially for older people with Long-term care (LTC) needs. It seems moreover crucial to enhance the overall system of social protection, especially the territorial public welfare, and to recognize and valorize the role of family caregivers, to be supported and fully integrated in the welfare and LTC system. For this purpose, an effective collaboration between formal and informal care should be pursued, in order to sustain also informal networks (including neighbours, friends, volunteering), especially in emergency times, thus making the whole care system more resilient [43]. All this to be realised with interventions close to the places where the most vulnerable older people live, ideally by means of a “zero km welfare”, in which public/formal home care and social workers can be strengthened, reducing the burden of families, and creating an integrated mix of public-private proximity services, to ensure that frail seniors receive continuous support, especially those ageing alone in place [84]. In addition, a reform of the IA is needed, through a redefinition and graduation of the amounts (currently the same for all beneficiaries, even though some steps are moving in this direction with the approval of the “enabling law”—i.e. Law n. 33/2023—for the reform of care of older people) based on the concrete level of support needs and economic situations of beneficiaries [16]. The new health-social priorities emerged during the COVID-19 outbreak also require to normalize innovative approaches like distance support services and working activities (e.g. telehealth, telemedicine) in public health and social sectors, in order to facilitate older people accessing to services, especially for those with multimorbidity and living in remote/rural areas. In this respect, the further aspect of low digital skill of seniors needs appropriate solutions, e.g., computer literacy programs. The measures suggested above, even though to be supported by further surveys, might contribute to build an overall framework for supporting frail older people, but also a “pandemic-resistant strategy”, thus preventing a “further overburdening of informal care networks” [43] (p. 10) in the near future. What is proposed represents a challenging task for decision makers and service providers, and requires specific public policies and implementation of good practices, which also emerged during the pandemic.

## Conclusions

Over the past decade, ageing in place has been of growing interest among people, local communities, researchers and politicians. The place of living and ageing should be a human right, a personal decision, but maintaining autonomy at home when living alone depends on many social, economic, and environmental aspects, especially in the light of health-social priorities emerged during the COVID-19 infection. Even though our study was exploratory only, with a small sample that cannot be considered representative of the target population, findings provide insights regarding the negative impact of the lockdown on frail older people living alone at home, also in comparison with the survey at T1, thus confirming the first hypothesis regarding overall hard consequences for seniors in the period. This to a different extent in Ancona and Brescia, but however with features not supporting the second hypothesis, regarding possible greater difficulties of older people in meeting basic needs in the latter city, following the greater impact of the COVID-19. In Brescia indeed, despite the greater number of contagions and deaths, a greater overall stability and support of municipal home services emerged, whereas in Ancona mainly family members were of help for daily needs of seniors, and access to health services was more difficult too. The health emergency, due to the COVID-19 pandemic, has therefore highlighted, in some way, the risks deriving from the various regional capabilities to face the health crisis and the consequent welfare inequalities [103], resulting in different possibilities, for the seniors, to react and manage their needs. Findings overall suggest that enhancing home care services, and supporting older people in having an adequate access to health services [93, 107], could allow ageing in place and give relief to family caregivers, especially in emergency times. This, also following the concept of the home as the first place of care for ageing people, to be supported with the strengthening of home care services, as stressed in the recent National Recovery and Resilience Plan (NRRP) for Italy [108]. Our small study has open space for further investigations, to obtain a complete picture of the issue under scrutiny. Larger follow-ups could highlight further useful insights for policy makers, in particular for an overall health promotion of older people, in order to support effectively their safe ageing in place without ageing alone.

## Supporting information

S1 Checklist STROBE statement: Checklist of items that should be included in reports of cross-sectional studies. (DOCX)

S1 Appendix Alphabetical list of abbreviations. (PDF)

S2 Appendix Survey questionnaire used in the follow-up study. (PDF)

## References

[1] Rogers WA, Ramadhani WA, Harris MT. Defining Aging in Place: The Intersectionality of Space, Person, and Time. Innov. Aging. 2020;4(4): igaa036. doi: 10.1093/geroni/igaa036 33173834 PMC7595274

[2] Wiles JL, Leibing A, Guberman N, Reeve J, Allen RES. The Meaning of “Aging in Place” to Older People. Gerontologist. 2012;52(3): 357–366. doi: 10.1093/geront/gnr098 21983126

[3] Djundeva M, Dykstra PA, Fokkema T. Is Living Alone "Aging Alone"? Solitary Living, Network Types, and Well-Being. J. Gerontol. B Psychol. Sci. Soc. Sci. 2019;74: 1406–1415. doi: 10.1093/geronb/gby119 30312447 PMC6777768

[4] Peace S, Holland C, Kellaher L. ‘Option recognition’ in later life: variations in ageing in place. Ageing Soc. 2011;31: 734–757. 10.1017/S0144686X10001157

[5] WHO. Creating age-friendly environments in Europe. A tool for local policy-makers and planners. Copenhagen, Denmark: WHO Regional Office for Europe; 2016. Available from: https://apps.who.int/iris/bitstream/handle/10665/334252/9789289052122-eng.pdf

[6] Grove H. Ageing as well as you can in place: Applying a geographical lens to the capability approach. Soc. Sci. Med. 2020;13: 113525. 10.1016/j.socscimed.2020.113525 33234454

[7] Pivetta NRS, Marincolo JCS, Neri AL, Aprahamian I, Yassuda MS, Borim FSA. Multimorbidity, frailty and functional disability in octogenarians: A structural equation analysis of relationship. Arch. Gerontol. Geriatr. 2020;86: 103931. doi: 10.1016/j.archger.2019.103931 31541858

[8] Cardoso AF, Bobrowicz-Campos E, Teixeira-Santos L, Cardoso D, Couto F, Apóstolo J. Validation and Screening Capacity of the European Portuguese Version of the SUNFRAIL Tool for Community-Dwelling Older Adults. Int. J. Environ. Res. Public Health. 2021;18: 1394. doi: 10.3390/ijerph18041394 33546251 PMC7913363

[9] Pilotto A, Custodero C, Maggi S, Polidori MC, Veronese N, Ferrucci L. A multidimensional approach to frailty in older people. Ageing Res. Rev. 2020;60: 101047. doi: 10.1016/j.arr.2020.101047 32171786 PMC7461697

[10] Wiseman RF. Why older people move. Res. Aging. 1980;22: 242–254. 10.1177/016402758022003

[11] Litwak E, Longino CF. Migration patterns among the elderly: A developmental perspective. Gerontologist. 1987;27(3): 266–272. doi: 10.1093/geront/27.3.266 3609792

[12] ISTAT. Popolazione Italiana residente al 1° Gennaio, 2022. Rome, Italy: ISTAT Geodemo; 2022. Available from: https://demo.istat.it/app/?l=it&a=2022&i=POS

[13] ISTAT. Aspetti Della Vita Quotidiana. Famiglie, Persone Sole. Rome, Italy: ISTAT; 2021. Available from: http://dati.istat.it/Index.aspx?QueryId=17631

[14] ISTAT. Le Condizioni di Salute Della Popolazione Anziana in Italia. Anno 2019. Rome, Italy: ISTAT Statistiche Report; 2021. Available from: https://www.istat.it/it/files//2021/07/Report-anziani-2019.pdf

[15] EPICENTRO–Epidemiology for Public Health. La sorveglianza Passi d’Argento. I dati per l’Italia. Fragilità e disabilità. Rome, Italy: EPICENTRO; 2020. Available from: https://www.epicentro.iss.it/passi-argento/dati/fragili#dati

[16] NNA—Network Non Autosufficienza, editor. L’assistenza agli anziani non autosufficienti in Italia. 7° Rapporto 2020/2021. Punto di non ritorno. Santarcangelo di Romagna, Italy: Maggioli; 2021. Available from: https://www.luoghicura.it/wp-content/uploads/2020/12/NNA_2020_7%C2%B0_Rapporto.pdf

[17] Barbabella F, Poli A, Chiatti C, Pelliccia L, Pesaresi F. La bussola di NNA: lo stato dell’arte basato sui dati. In: NNA—Network Non Autosufficienza, editor. L’assistenza agli anziani non autosufficienti in Italia, 6° Rapporto 2017/2018. Il tempo delle risposte. Santarcangelo di Romagna, Italy: Maggioli; 2017. pp. 33–54. Available from: https://www.luoghicura.it/wp-content/uploads/2017/12/NNA_2017_6%C2%B0_Rapporto.pdf

[18] Barbabella F, Poli A, Santini S, Lamura G. The role of informal caregivers in long-term care for older people: needs and supports. In: Boll T, Ferring D, Valsiner J, editors. Cultures of care: Handbook of cultural geropsychology. Charlotte NC, USA: Information Age Publishing; 2018. pp. 193–212.

[19] DOMINA. Quarto Rapporto annuale sul lavoro domestico. Analisi, statistiche, trend nazionali e locali. Venice, Italy: Osservatorio nazionale sul lavoro domestico, Fondazione Leone Moressa; 2022. Available from: https://www.osservatoriolavorodomestico.it/documenti/rapporto_annuale_2022.pdf

[20] Drożdżak Z, Melchiorre MG, Perek-Białas J, Principi A, Lamura G. Ageing and long-term care in Poland and Italy: a comparative analysis. In: Ervik R, Skogedal Lindén T, editors. The making of ageing policy: theory and practice in Europe. Cheltenham Glos, UK: Edwar Elgar Publishing Ltd; 2013. pp. 205–230.

[21] Melchiorre MG, Socci M, Quattrini S, Lamura G, D’Amen B. Frail Older People Ageing in Place in Italy: Use of Health Services and Relationship with General Practitioner. Int. J. Environ. Res. Public Health. 2022;19: 9063. doi: 10.3390/ijerph19159063 35897424 PMC9332283

[22] ISTAT. Anziani: Le condizioni di salute in Italia e nell’unione Europea. Anno 2015. Rome, Italy: ISTAT Statistiche Report; 2017. Available from: https://www.istat.it/it/files/2017/09/Condizioni_Salute_anziani_anno_2015.pdf

[23] Morrow-Howell N, Gonzales E. Recovering from Coronavirus Disease 2019 (COVID-19): Resisting Ageism and Recommitting to a Productive Aging Perspective. Public Policy Aging Rep. 2020;30: 133–137. 10.1080/08959420.2020.1759758 PMC7499785 38626253

[24] Kaufman BG, Whitaker R, Mahendraratnam N, Hurewitz S, Yi J, Smith VA, et al. State variation in effects of state social distancing policies on COVID-19 cases. BMC Public Health. 2021; 21: 1239. doi: 10.1186/s12889-021-11236-3 34182972 PMC8237534

[25] Lunn PD, Timmons S, Belton CA, Barjaková M, Julienne H, Lavin C. Motivating social distancing during the COVID-19 pandemic: An online experiment. Soc. Sci. Med. 2020;265: 113478. doi: 10.1016/j.socscimed.2020.113478 33162198

[26] Alfano V. Does social capital enforce social distancing? The role of bridging and bonding social capital in the evolution of the pandemic. Econ. Polit. 2022;39: 839–859. doi: 10.1007/s40888-021-00255-3 35422590 PMC8791696

[27] Seale H, Dyer CEF, Abdi I, Rahman KM, Sun Y, Qureshi MO, et al. Improving the impact of non-pharmaceutical interventions during COVID-19: examining the factors that influence engagement and the impact on individuals. BMC Infect. Dis. 2020;20(1): 607. doi: 10.1186/s12879-020-05340-9 32807087 PMC7430133

[28] Maloney W, Taskin T. Determinants of Social Distancing and Economic Activity During COVID-19: A Global View. Policy Research Working Paper 9242. Washington, DC: The World Bank; 2020. Available from: http://documents.worldbank.org/curated/en/325021589288466494/pdf/Determinants-of-Social-Distancing-and-Economic-Activity-during-COVID-19-A-Global-View.pdf

[29] Kantor BN, Kantor J. Non-pharmaceutical Interventions for Pandemic COVID-19: A Cross-Sectional Investigation of US General Public Beliefs, Attitudes, and Actions. Front. Med. 2020;7:384. doi: 10.3389/fmed.2020.00384 32719807 PMC7347901

[30] Xu H, Gan Y, Zheng D, Wu B, Zhu X, Xu C, et al. Relationship Between COVID-19 Infection and Risk Perception, Knowledge, Attitude, and Four Nonpharmaceutical Interventions During the Late Period of the COVID-19 Epidemic in China: Online Cross-Sectional Survey of 8158 Adults. J. Med. Internet. Res. 2020;22(11): e21372. doi: 10.2196/21372 33108317 PMC7669364

[31] Capodici A, Gori D, Lenzi J. Deaths, Countermeasures, and Obedience: How Countries’ Non-Pharmaceutical Measures Have Quelled the COVID-19 Death Toll. Front. Public Health. 2022;10: 934309. doi: 10.3389/fpubh.2022.934309 35812522 PMC9263288

[32] Cugmas M, Ferligoj A, Kogovšek T, Batagelj Z The social support networks of elderly people in Slovenia during the Covid-19 pandemic. PLoS ONE. 2021,16(3): e0247993. doi: 10.1371/journal.pone.0247993 33657172 PMC7928497

[33] Kostyál LÁ, Széman Z, Almási VE, Fabbietti P, Quattrini S, Socci M, et al. Impact of the COVID-19 Pandemic on Family Carers of Older People Living with Dementia in Italy and Hungary. Sustainability. 2021;13: 7107. 10.3390/su13137107

[34] Alfano V, Capasso S, Limosani M. On the determinants of anti-COVID restriction and anti-vaccine movements: the case of IoApro in Italy. Sci. Rep. 2023;13: 16784. doi: 10.1038/s41598-023-42133-x 37798271 PMC10556032

[35] Principi A, Lucantoni D, Quattrini S, Di Rosa M, Socci M. Changes in Volunteering of Older Adults in the Time of the COVID-19 Pandemic: The Role of Motivations. Int. J. Environ. Res. Public Health. 2022;19(22): 14755. doi: 10.3390/ijerph192214755 36429474 PMC9689984

[36] Tyrrell CJ, Williams KN. The paradox of social distancing: Implications for older adults in the context of COVID-19. Psychol. Trauma: Theory Res. Pract. Policy. 2020;12(S1): S214–S216. doi: 10.1037/tra0000845 32525379

[37] Daoust JF. Elderly people and responses to COVID-19 in 27 Countries. PLoS ONE. 2020;15(7): e0235590. doi: 10.1371/journal.pone.0235590 32614889 PMC7332014

[38] Tesch-Römer C, Lamura G. Older adults in the first wave of the Corona pandemic. Eur. J. Ageing. 2021;18: 145–147. doi: 10.1007/s10433-021-00629-3 34075314 PMC8154333

[39] OECD. Health at a Glance 2021: OECD Indicators. Paris, France: OECD Publishing; 2021. Available from: 10.1787/ae3016b9-en

[40] Rocard E, Sillitti P, Llena-Nozal A. COVID-19 in long-term care: Impact, policy responses and challenges. Paris, France: OECD Publishing; 2021. Available from: 10.1787/b966f837-en

[41] OCSE. State of Health in EU. Italia. Profilo della sanità 2021. Bruxelles, Belgium: OECD Publishing; 2021. Available from: https://health.ec.europa.eu/system/files/2021-12/2021_chp_it_italy.pdf

[42] Mathieu E, Ritchie H, Rodés-Guirao L, Appel C, Gavrilov D., Giattino C, et al. Coronavirus Pandemic (COVID-19). OurWorldInData.org; 2020. Available from: https://ourworldindata.org/coronavirus

[43] Tur-Sinai A, Bentur N, Fabbietti P, Lamura G. Impact of the Outbreak of the COVID-19 Pandemic on Formal and Informal Care of Community-Dwelling Older Adults: Cross-National Clustering of Empirical Evidence from 23 Countries. Sustainability. 2021;13(13): 7277. 10.3390/su13137277

[44] Commission European. State of Health in the EU. Companion Report 2021. Luxembourg: Publications Office of the European Union; 2021. Available from: https://eurohealthobservatory.who.int/publications/m/state-of-health-in-the-eu-companion-report-2021

[45] ISTAT-ISS. Impatto dell’epidemia COVID-19 sulla mortalità totale della popolazione residente. Anni 2020–2021 e Gennaio 2022. Rome, Italy: ISTAT; 2022. Available from: https://www.istat.it/it/files//2022/03/Report_ISS_ISTAT_2022_tab3.pdf

[46] Gauvin L, Bajardi P, Pepe E, Lake B, Privitera F, Tizzoni M. Socio-economic determinants of mobility responses during the first wave of COVID-19 in Italy: from provinces to neighbourhoods. J. R. Soc. Interface. 2021;18(181): 20210092. doi: 10.1098/rsif.2021.0092 34343450 PMC8331235

[47] Clegg A, Young J, Iliffe S, Rikkert MO, Rockwood K. Frailty in elderly people. Lancet. 2013;381(9868): 752–762. doi: 10.1016/S0140-6736(12)62167-9 23395245 PMC4098658

[48] Colombo F, Llena-Nozal A, Mercier J, Tjadens F. Help Wanted? Providing and Paying for Long-Term Care. Paris, France: OECD Publishing; 2011. Available from: https://read.oecd-ilibrary.org/social-issues-migration-health/help-wanted_9789264097759-en#page5

[49] ISTAT. Rapporto BES 2020. Il benessere equo e sostenibile in Italia. Rome, Italy: ISTAT; 2021. Available from: https://www.istat.it/it/files//2021/03/BES_2020.pdf

[50] Gioia E, Colocci A, Casareale C, Marchetti N, Marincioni F. The role of the socio-economic context in the spread of the first wave of COVID-19 in the Marche Region (central Italy). Int. J. Disaster Risk Reduct. 2022;82: 103324. doi: 10.1016/j.ijdrr.2022.103324 36213151 PMC9529353

[51] Rivieccio BA, Luconi E, Boracchi P, Pariani E, Romanò L, Salini S, et al. Heterogeneity of Covid-19 outbreak in Italy. Acta Biomed. 2020;91(2): 31–34. doi: 10.23750/abm.v91i2.9579 32420921 PMC7569642

[52] Cereda D, Manica M, Tirani M, Rovida F, Demicheli V, Ajelli M.; et al. The early phase of the COVID-19 epidemic in Lombardy, Italy. Epidemics. 2021;37: 100528. doi: 10.1016/j.epidem.2021.100528 34814093 PMC8605863

[53] Melchiorre MG, Quattrini S, Lamura G, Socci M. A Mixed-Methods Analysis of Care Arrangements of Older People with Limited Physical Abilities Living Alone in Italy. Int. J. Environ. Res. Public Health. 2021;18(24): 12996. doi: 10.3390/ijerph182412996 34948603 PMC8700972

[54] NSIA–National Strategy for Inner Areas. Annual report on the national strategy for Inner Areas. Rome, Italy: NSIA; 2018. Available from: https://www.agenziacoesione.gov.it/wp-content/uploads/2020/07/Relazione_CIPE_2018.pdf

[55] ISTAT. Le misure della vulnerabilità: un’applicazione a diversi ambiti territoriali. Rome, Italy: ISTAT; 2020. Available from: https://www.istat.it/it/files//2020/12/Le-misure-della-vulnerabilita.pdf

[56] Ritchie J, Lewis J, editors. Qualitative Research Practice. A Guide for Social Science Students and Researchers. London, UK: Sage Publications; 2003.

[57] Lamura G, Dohner H, Kofhal C, editors. Supporting Family Carers of Older People in Europe–Empirical Evidence, Policy Trends and Future Perspectives. Hamburg, Germany: Lit Verlag; 2008.

[58] Katz S. Assessing Self-Maintenance: Activities of Daily Living, Mobility, and Instrumental Activities of Daily Living. J. Am. Geriatr. Soc. 1983;31: 721–727. doi: 10.1111/j.1532-5415.1983.tb03391.x 6418786

[59] ISTAT. Indagine statistica multiscopo sulle famiglie; Scheda di rilevazione. Rome, Italy: ISTAT; 2011. Available from: https://www.istat.it/it/files//2011/01/Arancio_Mod_IMF_8A.pdf

[60] ISTAT. Conoscere il mondo della disabilità: persone, relazioni e istituzioni. Rome, Italy: ISTAT; 2019. Available from: https://www.istat.it/it/files//2019/12/Disabilita.pdf

[61] European Union. Regulation 2016/679 of the European parliament and of the Council. General data protection regulation. Off. J. Eur. Union. 2016;679: L 119/1. https://eur-lex.europa.eu/eli/reg/2016/679/oj

[62] Srivastava A, Thomson SB. Framework Analysis: A Qualitative Methodology for Applied Policy Research. J. Adm. Gov. 2009;4: 72–79. Available from: https://roam.macewan.ca:8443/server/api/core/bitstreams/53026c07-60e4-4bfc-b895-9d8e7e358b74/content

[63] Mayring P. Qualitative Content Analysis. Forum Qual. Soc. Res. 2000;1: 20. Available from: https://www.qualitative-research.net/index.php/fqs/article/view/1089/2385

[64] Gale NK, Heath G, Cameron E, Rashid S, Redwood S. Using the Framework Method for the Analysis of Qualitative Data in Multi-Disciplinary Health Research. BMC Med. Res. Methodol. 2013;13: 117. doi: 10.1186/1471-2288-13-117 24047204 PMC3848812

[65] Saldana J. The coding manual for qualitative researchers. London, UK: Sage Publications; 2009.

[66] Weitzman EA. Software and qualitative research. In: Denzin NK, Lincoln YS, editors. Handbook of Qualitative Research, 2nd ed. Thousand Oaks, CA, USA: Sage Publications; 2000. pp. 803–820.

[67] Chi MTH. Quantifying Qualitative Analyses of Verbal Data: A Practical Guide. J. Learn. Sci. 1997;6: 271–315. 10.1207/s15327809jls0603_1

[68] Kaplan S. Mixing quantitative and qualitative research. In: Elsbach K, Kramer R, editors. Handbook of Qualitative Organizational Research: Innovative Pathways and Methods. New York, USA: Routledge; 2016. pp. 423–433.

[69] Cerea S, Melchiorre MG. L’ageing in place alla prova della pandemia. Gli effetti indiretti del COVID-19 sugli anziani di Brescia e Ancona. DAStU Work. Pap. Ser. 2021;5: LPS.19. Available from: http://www.lps.polimi.it/wp-content/uploads/2021/05/DAStU_WP_n.-052021-LPS.19.pdf

[70] ISTAT-ISS. Impatto dell’epidemia Covid-19 sulla mortalità totale della popolazione residente. Gennaio-Maggio 2020. Rome, Italy: ISTAT; 2020. Available from: http://www.quotidianosanita.it/allegati/allegato3102684.pdf

[71] Brescia Today. Coronavirus Brescia: decessi, fasce d’età, patologie pregresse al 1st aprile 2020. Le statistiche dettagliate dall’inizio della pandemia. Brescia, Italy: Brescia Today; 2020. Available from: https://www.bresciatoday.it/attualita/coronavirus/eta-media.html

[72] Marche Region-GORES. Coronavirus Marche: Dati Servizio Sanità. Aggiornamento 1 Aprile 2020. Ancona, Italy: Marche Region-GORES; 2020. Available from: https://www.regione.marche.it/portals/0/Salute/CORONAVIRUS/DatiGORES/REPORT_sint_DECESSI_COVID19_aggiorn_1_APRILE_ore18.pdf

[73] Grondal M, Ask K, Luke TJ, Winblad S. Self-reported impact of the COVID-19 pandemic, affective responding, and subjective well-being: A Swedish survey. PLoS ONE. 2021;16(10): e0258778. doi: 10.1371/journal.pone.0258778 34653222 PMC8519461

[74] ISS–Istituto Superiore di Sanità. Protezione dei dati personali nell’emergenza COVID-19. Rapporto ISS COVID-19, n. 42/Maggio. Rome, Italy: ISS; 2020. Available from: https://www.iss.it/documents/20126/0/Rapporto+ISS+COVID-19+42_2020+%281%29.pdf/7fbd7a22-ba86-e323-1ff8-ebe2d4ae5da9?t=1608041817126

[75] ISTAT. Indagine “Inclusione sociale delle persone con limitazioni funzionali”—Anno 2011. Rome, Italy: ISTAT Nota metodologica; 2011. Available from: https://www.istat.it/it/files//2012/12/Nota-metodologica.pdf

[76] Maxwell JA. Using Numbers in Qualitative Research. Qual. Inq. 2010;16(6): 475–482. 10.1177/1077800410364740

[77] ISTAT. Reazione dei cittadini al lockdown, 5–21 Aprile 2020. Rome, Italy: ISTAT Statistiche Report; 2020. Available from: https://www.istat.it/it/files//2020/05/Reazione_cittadini_lockdown.pdf

[78] Age Platform Europe. COVID-19 and human rights concerns for older persons. Bruxelles, Belgium: Age Platform Europe; 2020. Available from: https://www.age-platform.eu/sites/default/files/Human_rights_concerns_on_implications_of_COVID-19_to_older_persons_updated_18May2020.pdf

[79] Monteduro G, Nanetti S. Invecchiamento, anziani fragili e Covid: una chiave di lettura. I luoghi della cura online. 2021;1: 1–7. Available from: https://www.luoghicura.it/sistema/cultura-e-societa/2021/02/invecchiamento-anziani-fragili-e-covid-una-chiave-di-lettura/?pdf

[80] Ooi L, Paul E, Burton A, Fancourt D, McKinlay AR. A qualitative study of positive psychological experiences and helpful coping behaviours among young people and older adults in the UK during the COVID-19 pandemic. PLoS ONE. 2023;18(1): e0279205. doi: 10.1371/journal.pone.0279205 36689484 PMC9870142

[81] Banchero A. Il Piano Nazionale della Cronicità in Italia. I luoghi della cura online. 2020;3: 1–7. Available from: https://www.luoghicura.it/sistema/programmazione-e-governance/2020/07/il-piano-nazionale-della-cronicita-in-italia/?pdf

[82] Costa G, Melchiorre MG, Arlotti M. Ageing in place in different care regimes. The role of care arrangements and the implications for the quality of life and social isolation of frail older people. DAStU Work. Pap. Ser. 2020;3: LPS.10. Available from: http://www.lps.polimi.it/wp-content/uploads/2020/09/WP-Dastu-32020_new-2.pdf

[83] Berloto S, Longo F, Notarnicola E, Perobelli E, Rotolo A. Il settore sociosanitario per gli anziani a un bivio dopo l’emergenza Covid-19: criticità consolidate e prospettive di cambiamento. In: Università Bocconi, CERGAS, editor. Rapporto OASI. Osservatorio sulle Aziende e sul Sistema sanitario Italiano. Milan, Italy: Egea Editore; 2020. pp. 203–236.

[84] Grusol—Gruppo Solidarietà, editor. Non come prima. L’impatto della pandemia nelle Marche. Moie di Maiolati, Italy: Grusol; 2021. Available from: http://www.grusol.it/apriSocialeN.asp?id=1037

[85] Marche Region. Ordinanza n. 19 del 3 aprile 2020. Chiusura Centri diurni sociosanitari. Ancona, Italy: Marche Region; 2020. Available from: https://www.regione.marche.it/Portals/0/Salute/Coronavirus/Regione%20Marche_Ordinanza%20COVID_19_n%2019%20del%203%20aprile%202020-signed.pdf

[86] Marche Region. Circolare esplicativa. Misure urgenti di contenimento del contagio sul territorio nazionale a seguito DPCM 9 marzo 2020. Ancona, Italy: Marche Region; 2020. Available from: https://www.regione.marche.it/Portals/0/Salute/Coronavirus/Regione%20Marche_circolare%20esplicativa%2011%20%20marzo%202020_COVID-19.pdf

[87] CronacheAncona.it. Coronavirus. L’assistenza domiciliare è garantita. Ancona, Italy: CronacheAncona.it; 2020. Available from: https://www.cronacheancona.it/2020/03/07/coronavirus-lassistenza-domiciliare-e-garantita/225766/

[88] Melzi A. Covid-19. Politiche sociali e sociosanitarie Lombarde di fronte all’emergenza. Milan, Italy: Vademecum Lombardia Sociale; 2020. Available from: http://www.lombardiasociale.it/wp-content/uploads/2020/09/Vademecum-20_Emergenza-Covid.pdf

[89] Nanetti S, Monteduro G, Moscatelli M. Gli anziani fragili e l’emergenza COVID-19: elementi d’innovazione nel welfare locale. Riv. Trim. Scienza Amm. 2020;2: 1–24. 10.32049/RTSA.2020.2.16

[90] Savla J, Robert KA, Blieszner R, McCann BR, Hoyt E, Knight AL. Dementia caregiving during the “stay-at-home” phase of COVID-19 pandemic. J. Gerontol. B Psychol. Sci. Soc. Sci. 2021;76: e241–e245. doi: 10.1093/geronb/gbaa129 32827214 PMC7546083

[91] Thoits PA. Mechanisms linking social ties and support to physical and mental health. J. Health Soc. Behav. 2011;52: 145–161. doi: 10.1177/0022146510395592 21673143

[92] Ferraro KF, Shippee TP, Schafer MH. Cumulative inequality theory for research on aging and the life course. In: Bengtson VL, Gans D, Putney NM, Silverstein M, editors. Handbook of theories of aging. New York, USA: Springer; 2009. pp. 413–433.

[93] Bardin A, Buja A, Barbiellini Amidei C, Paganini M, Favaro A, Saia M, et al. Elderly People’s Access to Emergency Departments during the COVID-19 Pandemic: Results from a Large Population-Based Study in Italy. J. Clin. Med. 2021;10: 5563. doi: 10.3390/jcm10235563 34884265 PMC8658732

[94] Lau VI, Dhanoa S, Cheema H, Lewis K, Geeraert P, Lu D, et al. Non-COVID outcomes associated with the coronavirus disease-2019 (COVID-19) pandemic effects study (COPES): A systematic review and meta-analysis. PLoS ONE. 2022;17(6): e0269871. doi: 10.1371/journal.pone.0269871 35749400 PMC9231780

[95] Mandrola J. CoViD-19 e dispositivi di protezione individuale: qualcuno di noi morirà per la loro carenza. Recenti Prog. Med. 2020;111(4): 183. 10.1701/3347.33175 32319434

[96] Cittadinanzattiva. XXIII Rapporto PIT Salute. Rome, Italy: Cittadinanzattiva; 2020. Available from: https://www.cittadinanzattiva.it/multimedia/import/files/primo_piano/salute/XXIII-Rapporto-PiT-Salute-2020-abstract.pdf

[97] Kuper H, Shakespeare T. Are older people with disabilities neglected in the COVID-19 pandemic? Lancet Public Health. 2021;6(6): e347–e348. doi: 10.1016/S2468-2667(21)00077-3 33894139 PMC8517572

[98] Genova A, Favretto AR, Clemente C, Servetti D, Lombardini S. Assistenza primaria e Covid-19: MMG e USCA. In: Vicarelli G, Giarelli G, editors. Il Servizio Sanitario Nazionale e la pandemia da Covid-19. Problemi e proposte. Milan, Italy: Franco Angeli; 2021. pp. 58–66. Available from: https://www.torrossa.com/it/resources/an/4925803#

[99] Cipolletta S, Previdi S, Martucci S. The Healthcare Relationship during the Second Wave of the COVID-19 Pandemic: A Qualitative Study in the Emergency Department of an Italian Hospital. Int. J. Environ. Res. Public Health. 2023;20: 2072. doi: 10.3390/ijerph20032072 36767436 PMC9916165

[100] Giarelli G, Vicarelli G. Conclusioni. Una bussola per il rilancio del SSN. In: Vicarelli G, Giarelli G, editors. Il Servizio Sanitario Nazionale e la pandemia da Covid-19. Problemi e proposte. Milan, Italy: Franco Angeli; 2021. pp. 117–141. Available from: https://www.torrossa.com/it/resources/an/4925803#

[101] Spandonaro F, D’Angela D, editors. Una misura di performance dei SSR, V edizione. Rome, Italy: CREA Sanità; 2017. Available from: https://careonline.it/wp-content/uploads/2017/07/Libro-Performance-V-Edition.pdf

[102] EURISPES. 32° Rapporto Italia, Percorsi di ricerca nella società italiana. Bologna, Italy: Minerva; 2020. Available from: https://www.cisge.it/ojs/index.php/geostorie/article/view/676

[103] Vicarelli G. Regionalismo sanitario e Covid-19: punti di forza e di debolezza. In: Vicarelli G, Giarelli G, editors. Il Servizio Sanitario Nazionale e la pandemia da Covid-19. Problemi e proposte. Milan, Italy: Franco Angeli; 2021. pp. 23–30. Available from: https://www.torrossa.com/it/resources/an/4925803#

[104] Arlotti M.; Marzulli M. La Regione Lombardia nella crisi sanitaria da Covid-19: ospedali, territorio e RSA. In: Vicarelli G, Giarelli G, editors. Il Servizio Sanitario Nazionale e la pandemia da Covid-19. Problemi e proposte. Milan, Italy: Franco Angeli; 2021. pp. 41–48. Available from: https://www.torrossa.com/it/resources/an/4925803#

[105] Skene SS, Kenward MG. The analysis of very small samples of repeated measurements. An adjusted sandwich estimator. Stat. Med. 2010;29(27): 2825–2837. 10.1002/sim.4073 20839367

[106] Polit DF, Beck CT. Generalization in Quantitative and Qualitative Research: Myths and Strategies. Int. J. Nurs. Studies. 2010;47: 1451–1458. doi: 10.1016/j.ijnurstu.2010.06.004 20598692

[107] Bashier H, Ikram A, Khan MA, Baig M, Al Gunaid M, Al Nsour M, et al. The Anticipated Future of Public Health Services Post COVID-19: Viewpoint. JMIR Public Health Surveill. 2021;7(6): e26267. doi: 10.2196/26267 33592576 PMC8216329

[108] Italian Presidency of the Council of Ministers. National Recovery and Resilience Plan (NRRP), #Nextgenerationitalia. Rome, Italy: Italian Presidency of the Council of Ministers; 2021. Available from: https://www.camera.it/temiap/2021/06/25/OCD177-4986.pdf

