# Supplementary material for: The impact of the COVID-19 pandemic on frail older people ageing in place alone in two Italian cities: Functional limitations, care arrangements and available services
Source: PLoS One. 2024 Mar 15;19(3):e0298074. doi: 10.1371/journal.pone.0298074 (PMC10942073; doi:10.1371/journal.pone.0298074)
Supplement: S1 Checklist — (DOCX) [file pone.0298074.s001.docx]

STROBE Statement—checklist of items that should be included in reports of observational studies

|  | | | | Item No. | | Recommendation | | Page  No. | Relevant text from manuscript |  |
| --- | --- | --- | --- | --- | --- | --- | --- | --- | --- | --- |
| **Title and abstract** | | | | 1 | | (*a*) Indicate the study’s design with a commonly used term in the title or the abstract | | 2 | Follow-up study (in the Abstract) |  |
|  |  |  |  |  |  | (*b*) Provide in the abstract an informative and balanced summary of what was done and what was found | | 2 | The study aimed to explore and compare effects of lockdown, due to the COVID-19 pandemic in 2020, on frail older people living alone at home in Brescia and Ancona, two urban cities located respectively in Northern and Central Italy. A follow-up study of the IN-AGE research project (2019) was carried out in July-September 2020, by means of telephone interviews, involving 41 respondents. The lockdown and social distancing measures overall negatively impacted on frail older people living alone, to a different extent in Ancona and Brescia, with a better resilience of home care services in Brescia, and a greater support from the family in Ancona. |  |
| Introduction | | | | | | | | |  |  |
| Background/rationale | | | | 2 | | Explain the scientific background and rationale for the investigation being reported | | 3-6 | A poor and/or inadequate care and cure network could make it difficult for seniors to remain at home, especially in a context of health emergency, as the one following the COVID-19 outbreak and the consequent lockdown. In Italy the national lockdown allowed to go out of one’s house only for working, for reasons regarding health, and to purchase primary goods [29]. However, this precaution drastically reduced social interactions, especially with family members/relatives, friends, and neighbours, with hard consequences in particular on well-being of older people. |  |
| Objectives | | | | 3 | | State specific objectives, including any prespecified hypotheses | | 6-7 | 1) Did the health emergency during the first wave of the COVID-19 pandemic impact on older people living alone, with regard to their functional limitations, e.g., mobility and other activities of daily living? 2) Did the pandemic impact on available care networks and services, e.g., family, friends and neighbours, municipal social services (e.g., SAD), DHH, PCA, and volunteering? 3) Did the health emergency due to the COVID-19 outbreak impact on access and use to/of health services? 4) Were there differences/similarities between the cities of Ancona and Brescia in this regard? |  |
| Methods | | | | | | | | |  |  |
| Study design | | | | 4 | | Present key elements of study design early in the paper | | 7-9 | The main survey, carried out in 2019, was a qualitative research project that involved 120 older people. In three Italian regions (Lombardy in the North, Marche in the Centre, and Calabria in the South), respectively three medium-sized urban cities [12] (with 100,000-200,000 inhabitants, i.e., Brescia, Ancona, and Reggio Calabria), and three inner/rural areas [47] were examined (respectively: Oltrepò Pavese, Appennino Basso Pesarese Anconetano, and Area Grecanica). The follow-up study was carried out in July-September 2020, in Brescia (Lombardy region) and Ancona (Marche region), with the aim of collecting experiences of older people living alone (i.e., without cohabiting relatives) in particular during the first wave of COVID-19 pandemic (February-May 2020), in relation to some dimensions investigated also in 2019. |  |
| Setting | | | | 5 | | Describe the setting, locations, and relevant dates, including periods of recruitment, exposure, follow-up, and data collection | | 10 | Twenty and 21 older people were interviewed respectively in Brescia and Ancona in 2020, compared to 24 in both cities during the previous survey in 2019. Respondents were re-contacted with the help of recruitment channels involved in the main research project (local branches of voluntary associations and operators of municipal social services, in particular home care, i.e., SAD). Interviews were realized by telephone due to social distancing imposed by the pandemic. Thus they were not audio-recorded, but answers were written on papers by interviewers. The questionnaire (S2 Appendix) was semi-structured with closed-ended questions, and the possibility however to specify/support the answers by means of free/open spaces in the questionnaire itself was also provided. |  |
| Participants | | | | 6 | | (*a*) *Cohort study*—Give the eligibility criteria, and the sources and methods of selection of participants. Describe methods of follow-up  *Case-control study*—Give the eligibility criteria, and the sources and methods of case ascertainment and control selection. Give the rationale for the choice of cases and controls  ***Cross-sectional study*—Give the eligibility criteria, and the sources and methods of selection of participants** | | 7 | (For the main IN-AGE cross-sectional study) A purposive sampling was provided [49], and frail older persons were recruited through the local branches of a major volunteering organisation. The inclusion criteria were the following: men and women aged 65 years and over, living alone at home or with the support of a PCA, limited functionalities/mobility, absence of cognitive impairment, and absence of very close family members who give them help. |  |
|  |  |  |  |  |  | (*b*) *Cohort study*—For matched studies, give matching criteria and number of exposed and unexposed  *Case-control study*—For matched studies, give matching criteria and the number of controls per case | |  |  |  |
| Variables | | | | 7 | | Clearly define all outcomes, exposures, predictors, potential confounders, and effect modifiers. Give diagnostic criteria, if applicable | | 10-11 | Questions addressed first of all the impact of COVID-19 on health, e.g., having experienced or not a contagion. In addition, self-reported changes (compared to 2019) due to lockdown were explored with regard to the following aspects: overall functional limitations, as mobility and ability to carry out the activities of daily living; available care networks/arrangements; and access/use to/of health services (GP, MS, other health services). |  |
| Data sources/ measurement | | | | 8* | | For each variable of interest, give sources of data and details of methods of assessment (measurement). Describe comparability of assessment methods if there is more than one group | | 11 | It was decided to detect self-reported impact of the COVID-19 outbreak in order to catch the perceived changes on different domains of participants’ lives, by asking older people to report possible worsening/improving due to the pandemic. |  |
| Bias | | | | 9 | | Describe any efforts to address potential sources of bias | | 30 | Our study was exploratory only, with a small sample that cannot be considered representative of the target population. |  |
| Study size | | | | 10 | | Explain how the study size was arrived at | | 2; 9-10 | A follow-up study of the IN-AGE research project (2019) was carried out in July-September 2020, by means of telephone interviews, involving 41 respondents.  The follow-up study was carried out in July-September 2020, in Brescia (Lombardy region) and Ancona (Marche region), with the aim of collecting experiences of older people living alone (i.e., without cohabiting relatives) in particular during the first wave of COVID-19 pandemic.  Twenty and 21 older people were interviewed respectively in Brescia and Ancona in 2020, compared to 24 in both cities during the previous survey in 2019. |  |
| Continued on next page Quantitative variables | | 11 | | | Explain how quantitative variables were handled in the analyses. If applicable, describe which groupings were chosen and why | | 11-13 | | Functional limitations and care arrangements for T2 have been explored only as changes referred directly by respondents compared to T1. The access to health services has been analysed comparing the use referred at T1 with regard to GP, MS, and other health services, with possible different/worsened access to these three services at T2, as referred by respondents. | |
| Statistical methods | | 12 | | | (*a*) Describe all statistical methods, including those used to control for confounding | | 11 | | A simple quantitative analysis (frequency distribution/bivariate analysis) of closed responses was carried out by using Microsoft Excel software 2019. | |
|  |  |  |  |  | (*b*) Describe any methods used to examine subgroups and interactions | | N.A. | |  | |
|  |  |  |  |  | (*c*) Explain how missing data were addressed | | 12; 30 | | Several empty spaces in the tables reflect the choose to show only the answers of participants referring changes.  Our study was exploratory only, with a small sample that cannot be considered representative of the target population. | |
|  |  |  |  |  | (*d*) *Cohort study*—If applicable, explain how loss to follow-up was addressed  *Case-control study*—If applicable, explain how matching of cases and controls was addressed  ***Cross-sectional study*—If applicable, describe analytical methods taking account of sampling strategy** | | N.A. | |  | |
|  |  |  |  |  | (*e*) Describe any sensitivity analyses | | N.A. | |  | |
| Results | | | | | | | | | | |
| Participants | | 13* | | | (a) Report numbers of individuals at each stage of study—eg numbers potentially eligible, examined for eligibility, confirmed eligible, included in the study, completing follow-up, and analysed | | 7; 10 | | The main survey, carried out in 2019, was a qualitative research project that involved 120 older people.  Twenty and 21 older people were interviewed respectively in Brescia and Ancona in 2020, compared to 24 in both cities during the previous survey in 2019. | |
|  |  |  |  |  | (b) Give reasons for non-participation at each stage | | 13 | | The follow-up could not include only four subjects in Brescia (three deceased before the start of the follow-up in July 2020, and one refusal to be interviewed) and three in Ancona (one deceased and two refusals). | |
|  |  |  |  |  | (c) Consider use of a flow diagram | | 30 | | Our study was exploratory only, with a small sample. | |
| Descriptive data | | 14* | | | (a) Give characteristics of study participants (eg demographic, clinical, social) and information on exposures and potential confounders | | 13-14 | | Overall, the following prevail: older people aged 85 and over (especially in Ancona), women, with a low educational level, widowers (especially in Ancona), and living alone without PCA (the total in Brescia) (Table 1). | |
|  |  |  |  |  | (b) Indicate number of participants with missing data for each variable of interest | | 15-17; 20 | | Tables 2, 3, 4. | |
|  |  |  |  |  | (c) *Cohort study*—Summarise follow-up time (eg, average and total amount) | |  | |  | |
| Outcome data | | 15* | | | *Cohort study*—Report numbers of outcome events or summary measures over time | |  | |  | |
|  |  |  |  |  | *Case-control study—*Report numbers in each exposure category, or summary measures of exposure | |  | |  | |
|  |  |  |  |  | ***Cross-sectional study—*Report numbers of outcome events or summary measures** | | 15-17; 20 | | Tables 2, 3, 4. | |
| Main results | | 16 | | | (*a*) Give unadjusted estimates and, if applicable, confounder-adjusted estimates and their precision (eg, 95% confidence interval). Make clear which confounders were adjusted for and why they were included | | 14-22 | | Findings showed overall that seniors had a stronger support from home care services in Brescia, and a greater support from the family members in Ancona, where access to health services emerged as more problematic. The comparison between the two urban contexts also partly highlighted regional welfare inequalities in Italy which impact on ageing in place. | |
|  |  |  |  |  | (*b*) Report category boundaries when continuous variables were categorized | | 11; 13; 15 | | Physical/functional limitations have been classified for T1 as follows [68]: mild: no activity ‘not able’; moderate: one-two; high: three-four; very high: five and more (Table 2)  Age group (Table 1): 65-74, 75-79, 80-84. | |
|  |  |  |  |  | (*c*) If relevant, consider translating estimates of relative risk into absolute risk for a meaningful time period | | N.A. | |  | |
| Continued on next page Other analyses | 17 | | Report other analyses done—eg analyses of subgroups and interactions, and sensitivity analyses | | | | | 10; 12-13; 15-22 | The questionnaire (S2 Appendix) was semi-structured with closed-ended questions, and the possibility however to specify/support the answers by means of free/open spaces in the questionnaire itself was also provided.  Open responses were not categorized since they were really few, fragmented, and overall supporting the closed answers. However, along the results section, some short quotations have been included when relevant (where BS stands for Brescia, and AN for Ancona). Further details have been added within the text, by means of simple quantifications/count of things, when reported as spontaneous/not asked narratives [69], even though by few interviewees, with the aim to include other information integrating the overall findings, e.g., worsening due to reduced/suspended SAD/MS visits; specific family members (e.g., daughter) providing particular help during the lockdown. | |
| Discussion | | | | | | | | | | |
| Key results | 18 | | Summarise key results with reference to study objectives | | | | | 22 | Findings showed overall that seniors had a stronger support from home care services in Brescia, and a greater support from the family members in Ancona, where access to health services emerged as more problematic. The comparison between the two urban contexts also partly highlighted regional welfare inequalities in Italy which impact on ageing in place. | |
| Limitations | 19 | | Discuss limitations of the study, taking into account sources of potential bias or imprecision. Discuss both direction and magnitude of any potential bias | | | | | 28-30 | The paper is based on a small sample size (total N=41), that cannot be considered representative of the target population, thus allowing only simple/general conclusions and purposes from the study, rather than politically relevant insights.  The interviews were short and carried out by telephone, since face-to-face administration was prevented during the lockdown due to the social distancing imposed by the pandemic and related government restrictions, without the possibility to collect detailed audio-recorded narratives as for T1 (replaced at T2 by written responses/notes that interviewers filled-in during the short call phone).  Furthermore, the overall inclusion/comparison of data at different territorial levels (e.g., municipal with regard to results of follow-up, provincial with regard to infections and deaths for COVID-19, and also regional/national/international for their discussion), is sometimes depending on the paucity of information provided by available sources, which were however mainly in Italian, especially when regarding local data. For this reason, the manuscript contains several references in this language.  The definition of frailty is limited to old age (65 years and over), ageing alone in place, and presence of functional limitations needing support in the activities of daily living; the cognitive assessment of interviewees was based on the information from the recruitment channels, then confirmed by the respective families. | |
| Interpretation | 20 | | Give a cautious overall interpretation of results considering objectives, limitations, multiplicity of analyses, results from similar studies, and other relevant evidence | | | | | 30-31 | Maintaining autonomy at home when living alone depends on many social, economic, and environmental aspects, especially in the light of health-social priorities emerged during the COVID-19 infection. Findings provides insights regarding the negative impact of the lockdown on frail older people living alone at home, also in comparison with the survey at T1, thus confirming the overall hard consequences for seniors in the period. This to a different extent in Ancona and Brescia. The health emergency, due to the COVID-19 pandemic, has therefore highlighted, in some way, the risks deriving from the various regional capabilities to face the health crisis and the consequent welfare inequalities [96], resulting in different possibilities, for the seniors, to react and manage their needs. | |
| Generalisability | 21 | | Discuss the generalisability (external validity) of the study results | | | | | 29-30 | (In) The survey carried out in 2019/T1 (…) subjects were selected for their typological and not statistical representativeness, since the study was more qualitative. (The follow-up) was exploratory only, with a small sample that cannot be considered representative of the target population. | |
| Other information | | |  | | | | | | | |
| Funding | 22 | | Give the source of funding and the role of the funders for the present study and, if applicable, for the original study on which the present article is based | | | | |  | This is provided and detailed in the related space (Financial disclosure) during the on-line submission process: The paper was produced within the framework of the IN-AGE project, funded by Fondazione Cariplo ([https://www.fondazionecariplo.it/it/index.html#](https://www.fondazionecariplo.it/it/index.html)), Grant N. 2017-0941. The Project was awarded to GL. This work has also partially been supported by the Ricerca Corrente funding from the Italian Ministry of Health to IRCCS INRCA. The funders had no role in study design, data collection and analysis, decision to publish, or preparation of the manuscript. | |

*Give information separately for cases and controls in case-control studies and, if applicable, for exposed and unexposed groups in cohort and cross-sectional studies.

**Note:** An Explanation and Elaboration article discusses each checklist item and gives methodological background and published examples of transparent reporting. The STROBE checklist is best used in conjunction with this article (freely available on the Web sites of PLoS Medicine at http://www.plosmedicine.org/, Annals of Internal Medicine at http://www.annals.org/, and Epidemiology at http://www.epidem.com/). Information on the STROBE Initiative is available at www.strobe-statement.org.
