# Supplementary material for: The impact of the COVID-19 pandemic on frail older people ageing in place alone in two Italian cities: Functional limitations, care arrangements and available services
Source: PLoS One. 2024 Mar 15;19(3):e0298074. doi: 10.1371/journal.pone.0298074 (PMC10942073; doi:10.1371/journal.pone.0298074)
Supplement: S1 Appendix — (PDF) [file pone.0298074.s002.pdf]

S1 - Alphabetical list of abbreviations

ADL	Activity of Daily Living
AUSER	Voluntary Association for Active Ageing
DHH	Domestic Home Helper
ERP	Public Housing (*Edilizia Residenziale Pubblica*)
EU	European Union
GDPR	General Data Protection Regulation
GP	General Practitioner
IA	National Disability Attendance Allowance (*Indennità di accompagnamento*)
IADL	Instrumental Activity of Daily Living
IN-AGE	Inclusive ageing in place
MCW	Migrant Care Worker
MS	Medical Specialist
NHS	National Health Service
NRRP	National Recovery and Resilience Plan
OWID	Our World in Data
PCA	Personal Care Assistant
POLIMI	Polytechnic of Milan
RHS	Regional Health System
SAD	Home Care Service (*Servizio di Assistenza Domiciliare*)
SHARE	Survey of Health Ageing and Retirement
T1	2019 Main IN-AGE survey
T2	2020 follow-up
