# Supplementary material for: The impact of the COVID-19 pandemic on frail older people ageing in place alone in two Italian cities: Functional limitations, care arrangements and available services
Source: PLoS One. 2024 Mar 15;19(3):e0298074. doi: 10.1371/journal.pone.0298074 (PMC10942073; doi:10.1371/journal.pone.0298074)
Supplement: S2 Appendix — (PDF) [file pone.0298074.s003.pdf]

S2 - Questionnaire [ID participant: BS/AN n..........]

Note to the interviewer: *the questions below in blue colour (COVID and T2/follow-up) are to be filled in. If changes are referred, ask the senior to explain briefly why/how the situation changed, and take note of the answer. The questions in black colour (T1) have to be completed with data from the survey carried out in 2019.*

Good morning/Good evening. Am I speaking to Mr. / Mrs. [NAME]?
My name is [NAME, ORGANIZATION]. I don't know if you remember, but last year, on [date of first interview], you kindly accepted to participate in the IN-AGE research project, and to be interviewed at your home. During the interview, you kindly told me about your daily life, your needs and supports, your health and relationships with family/friends, and so on. Your narrative was very important for understanding how older people live in our country today, especially those who live alone. Currently I am calling you again first of all to find out how you are doing, and then to ask a few questions about how you are living since the COVID-19 pandemic emergency began. I don't want take up too much of your time. You will be able to respond freely by thinking about your experience over the past few months, from February to today. If you agree to participate, all data collected will be stored anonymously, for scientific research purposes only, and will be used to better understand the consequences of this terrible pandemic for older people.

COVID
First of all, we would kindly ask if you have contracted the virus
- 1 = No
- 2 = Yes

Then, we would know if, as a result of the pandemic, your situation changed compared to 2019, regarding some aspects of your daily living, as explained below.

Age
- 1 = 65-74 years
- 2 = 75-79 years
- 3 = 80-84 years
- 4 = 85 and over

Gender
- 1 = Female
- 2 = Male

Living situation
- 1 = Alone
- 2 = With cohabitant Personal Care Assistant (PCA)
- 3 = With hourly Personal Care Assistant (PCA)

T2 Living situation
- 1 = Alone
- 2 = With cohabitant Personal Care Assistant (PCA)
- 3 = With hourly Personal Care Assistant (PCA)

T2 Change in the help from Personal Care Assistant (PCA)
- 1 = No change
- 2 = Yes, worsened (*e.g., previous PCA no more available*)

  ____

  ____

- 3 = Yes, improved (*e.g., a new PCA was hired; from hourly PCA to cohabitant PCA*)

  ____

  ____

Marital status

- 1 = Married but not cohabiting
- 2 = Divorced/separated
- 3 = Widowed
- 4 = Single

Education

- 1 = No title
- 2 = Primary school (5 years)
- 3 = Middle school (3 years)
- 4 = High school (3-5 years)
- 5 = University/similar (3-5 years)

Daily living activities: level of limitations

- 1 = Mild
- 2 = Moderate
- 3 = High
- 4 = Very high

T2 Change in overall mobility (ability to move in general, to walk**)**

- 1 = No change
- 2 = Yes, worsened

  ____

  ____

  _

T2 Change in other physical activities on the whole *(e.g., dressing/undressing, washing hands/face, bathing/showering, preparing/eating/cutting food, cleaning the house, washing the laundry, shopping, taking medication, managing finances)*

- 1 = No change
- 2 = Yes, worsened

  ____

  ____

Help from the family (*share of family help on the total help from family/friends/services*)

- 1 = No help
- 2 = Moderate (up to 50% of total help)
- 3 = Strong (> 50% of total help)

T2 Change in the help from the family

- 1 = No change
- 2 = Yes, worsened

  ____

  ____

- 3 = Yes, improved

  ____

  ____

Help from municipal social services

- 1 = No help
- 2 = Help from public home care (SAD)
- 3 = Help from day centre

T2 Change in the help from municipal social services

- 1 = No change
- 2 = Yes, worsened
    - SAD
    - Day centre
- 3 = Yes, improved
    - SAD
    - Day centre

Help from Domestic Home Helper (DHH)

- 1 = No help
- 2 = Yes

T2 Change in the help from Domestic Home Helper (DHH)

- 1 = No change
- 2 = Yes, worsened
- 3 = Yes, improved

Help from friends/neighbours

- 1 = No help
- 2 = Yes

T2 Change in the help from friends/neighbours

- 1 = No change
- 2 = Yes, worsened
- 3 = Yes, improved

Help from volunteering

- 1 = No help
- 2 = Yes

T2 Change in the help from volunteering

- 1 = No change
- 2 = Yes, worsened
- 3 = Yes, improved

T2 Change in support from/access to General Practitioner (GP) *(e.g., prescription of drugs, therapies, clinical tests, check-ups for pathologies already suffered or for the onset of new symptoms)*

- 1 = No change
- 2 = Yes, worsened

  ____

  ____

- 3 = Yes, different access

  ____

  ____

T2 Change in support from/access to Medical Specialist (MS) *(e.g., prescription of drugs, therapies, clinical tests, check-ups for pathologies already suffered or for the onset of new symptoms)*

- 1 = No change
- 2 = Yes, worsened access

  ____

  ____

- 3 = Yes, different access

  ____

  ____

  ____

T2 Change in support from/access to other health services *(e.g., nurse, rehabilitation/physiotherapy, diagnostic tests)*

- 1 = No change
- 2 = Yes, worsened

  ____

  ____

- 3 = Yes, different access

  ____

  ____
